# Temporal trends in hospital-recorded pulmonary embolism in England before, during and after the COVID-19 pandemic (2008–2024): a population-based observational study

**DOI:** 10.1016/j.lanepe.2025.101433

**Published:** 2025-09-02

**Authors:** Xiaomin Zhong, James Webster, Eva J.A. Morris, Emre Oguzman, Susan Shapiro, Sasha Shepperd, Raph Goldacre

**Affiliations:** a Applied Health Research Unit, Nuffield Department of Population Health, University of Oxford, Oxford, UK; b Department of Haematology, Oxford University Hospitals NHS Foundation Trust, Oxford, UK; c Radcliffe Department of Medicine, Oxford University, Oxford, UK

**Keywords:** Pulmonary embolism, COVID-19 pandemic, Temporal trend, Hospital admissions

## Abstract

**Background:**

COVID-19 infection increases the risk of pulmonary embolism (PE). Up-to-date reporting of hospitalisation rates for PE is needed to inform service planning and for benchmarking in light of the pandemic. Our primary aim was to quantify monthly trends in first-time, hospital-recorded PE across England from 2008 to 2024, with particular focus on the pandemic period. A secondary aim was to examine how these trends varied by age, sex, socioeconomic deprivation, and region, providing ongoing public access through an interactive online dashboard.

**Methods:**

We conducted an epidemiological population-based study of all first-time PE diagnoses using English national secondary care data from April 2008 to December 2024. Trends before and after the onset of the COVID-19 pandemic (March 2020) were compared, with analyses by age, sex, region, and deprivation.

**Findings:**

A total of 750,109 first-time PE admissions were identified. Age-standardised first-time hospital-recorded PE rates rose from 5.4 per 100,000 population in April 2008 to 8.5 in January 2020, spiked to 16.8 in January 2021 during the pandemic. The spike was largely accounted for by PEs where COVID-19 was a co-existing diagnosis. Rates have since declined, returning to pre-pandemic levels by early 2023 (e.g. March 2023, 8.6 per 100,000), and may be continuing to decline, subject to further updates. Regional and deprivation gradients persisted throughout but were more pronounced during the pandemic.

**Interpretation:**

Whilst incidence of hospital-recorded PE spiked during the COVID-19 pandemic, rates have since returned to levels observed immediately prior to the pre-pandemic. Whilst it is too early to determine whether the recent downward trend may begin to reverse some of the upward trend observed over the decade before the pandemic, continued surveillance of hospital-recorded PE reported via our online tool will keep these findings up to date. Ongoing monitoring of PE incidence by healthcare setting is important to undertake while clinical practice and policy on PE management pathways evolve, since it helps to support care planning; it also informs data-enabled clinical trials where PE is an outcome as well as the design of observational studies.

**Funding:**

This work was supported by the NIHR Biomedical Research Centre, Oxford and by 10.13039/501100023699Health Data Research UK.

**Research in context**

**Evidence before this study**

We searched PubMed for published population-based studies that reported on the incidence of pulmonary embolism (PE) from 1 January 2010 until 20 May 2025 using the search terms “pulmonary embolism” or “venous thromboembolism” and “incidence”, without restrictions on language or country. Previously published studies have reported a long-term increase in the incidence of PE in many countries over the decades 2000–2019, which has been attributed to an increased prevalence of risk factors for PE and improved access to more sensitive diagnostic imaging modalities. COVID-19 has been reported as a risk factor for PE, and PE incidence increased during the COVID-19 pandemic (2020–2021). However, accurate, large-scale, contemporary data on how PE incidence changed both during and since the COVID-19 pandemic are limited.

**Added value of this study**

This large-scale population-based study provides novel information on the incidence of hospital-recorded PE during and after the COVID-19 pandemic in England at the monthly level, with reference to long-term trends before the pandemic. Using multiple-cause coded person-based national hospital data, we were able to accurately ascertain all first-time hospital-recorded PEs from 2008 to 2024, including those where PE was recorded secondary to other conditions such as COVID-19. Following an upward long-term trend prior to the pandemic, age-standardised and age-specific population-based rates of first-time hospital-recorded PE spiked substantially during the pandemic in those with a co-existing diagnosis of COVID-19 infection. Rates returned to pre-pandemic levels by early 2023. Whilst it is too early to determine whether the recent downward trend may begin to reverse some of the consistent upward trend observed over the decade before the pandemic, continued surveillance of hospital-recorded PE reported via our online tool will keep these findings up to date, to inform evolving strategies for PE diagnosis, prevention and management.

**Implications of all the available evidence**

The COVID-19 pandemic, and COVID-19 infection itself, had a significant direct impact on the pattern of hospital-recorded PE rates. Having spiked significantly during the pandemic, rates since 2023 have returned to pre-pandemic levels. Ongoing monitoring of hospital-recorded PE, and further research including examining incidence by care pathway, is needed to track the success of prevention and management strategies, especially in the context of evolving policy guidelines, and in anticipation of potential future public health crises. Future updates to these analyses will continue to track these rates via an online interactive tool. Data from other countries that include the pandemic and post-pandemic periods are needed to clarify the generalisability of these trends and the impact on healthcare systems in other regions.

## Introduction

Pulmonary embolism (PE) is a significant global health issue affecting 1 in 10,000 people each year.^1^^,^^2^ Quality of life after PE is often diminished,^3^ around half of all patients diagnosed with PE have functional and exercise limitations a year later,^4^ and one in five patients who are treated for PE die within 90 days.^5^ The economic cost to health systems is estimated at 9100–10,600 euros in the first year following diagnosis.^3^ Preventing PE is therefore a priority for public health and health services. Hospital-based data are important for tracking PE trends and evaluating public health initiatives to prevent PE and treat it effectively, due to the reliable coding for hospital admissions for PE. PE prevention is also a key medical inpatient safety measure, further underlining the need for accurate and up to date reporting,^6^ but surveillance of PE incidence is not yet routine.

Infection with COVID-19 has been reported to be associated with a higher risk of PE.^2^^,^^7^, ^8^, ^9^, ^10^, ^11^, ^12^ The COVID-19 pandemic also disrupted the delivery of healthcare with potential indirect effects on the incidence and presentation of PE. For example, emergency admissions for acute cardiovascular events including PE decreased during the first wave, thought to be due to a change in health-seeking behaviour among the public for fearing of contracting COVID-19 in hospital.^13^ At the same time, elective surgical admissions such as hip and knee replacements that specifically increase the risk of developing PE in hospital operated at substantially reduced capacity.^14^ PE incidence has reportedly increased during the years covering the COVID-19 pandemic (2020–2021) in some countries.^2^^,^^7^^,^^8^^,^^10^^,^^11^ However, the available literature leaves important gaps, particularly in monthly, population-based trend data that cover both pandemic waves and the years that followed. The most recent study of national PE population-based diagnosis rates in hospitals in England reported an increase in the population-based rate of PE incidence from 2005 to 2022, with no significant change in 2020–2021 compared to the pre-pandemic trend.^2^ However, that study was based on unlinked aggregated annual hospital statistics using primary diagnoses only, and therefore was subject to under-counting of PE events where a co-occurring condition (notably COVID-19) was listed as the primary diagnosis.

Accurate and up-to-date reporting of hospital-recorded PE incidence before, during, and after the pandemic is needed to evidence-based planning, such as the allocation of healthcare resources, for pandemic preparedness, and for benchmarking of progress in preventing PE. This requires record linkage to distinguish first-time admissions and account for COVID-19 as a coexisting condition. Continued monitoring of PE incidence also helps to inform data-enabled clinical trials and observational studies where the condition is an outcome. Up-to-date information on PE incidence is particularly important in light of the systemic disruption to the health service caused by the COVID-19 pandemic,^15^ and other influences including evolving practices for PE prevention, diagnosis, and management.^16^^,^^17^

Using national record-linked hospital data in England covering over two decades, the objective of this study was to report a comprehensive set of monthly rates for new PE diagnoses in England from 2008 to 2024, assessing the impact of the pandemic on PE hospitalisation rates. A secondary aim was to explore variation in these trends by age, sex, deprivation, and region. Future updates to these analyses, reported via an interactive online tool, will continue to track these rates to monitor PE incidence across England.

## Methods

### Ethical approval

Under the assessment of the NHS Health Research Authority, using the HES APC data to conduct epidemiological and health service research at the University of Oxford does not need research ethics committee approval as it is anonymised data.

### Study design and data

We conducted a national, retrospective observational study of hospital-based diagnosis of PE across all NHS hospitals in England. The study period was from April 2008 to December 2024, ensuring a minimum five-year look-back for every participant so that only first-time hospital-recorded PE events were included. Data were derived from Hospital Episode Statistics Admitted Patient Care (HES-APC) records, which include a primary diagnosis and up to 19 secondary diagnoses per hospital episode, coded using the International Classification of Diseases, Tenth Revision (ICD-10).

### Study population

Patients were identified as having PE if the ICD-10 code I26 appeared in any diagnostic position. This approach to PE case ascertainment was based on previous literature and clinical coding recommendations.^18^, ^19^, ^20^, ^21^ Patients with a concurrent diagnosis of COVID-19 were identified using ICD-10 code U07.1 (or U07.2 in sensitivity analysis) in any diagnostic position.

### Outcomes

First-time PE-related hospital diagnosis counts were the primary outcome, defined as the first hospital-recorded PE per patient, in any diagnosis position, over the entire study period, ensuring a minimum five-year look-back for every individual using data back to April 2003, so that only first-time hospital-recorded PE events were included. This measure was selected as the most unbiased for assessing epidemiological trends, since second PE events (recurrence) cannot be reliably distinguished from re-recordings or follow-up care for earlier PEs.^22^ A minimum lookback of 5 years was chosen since analysis of the full HES-APC extract available from April 2003 indicated that only 3% of individuals with a hospital-recorded PE diagnosis in 2019 (i.e. the last pre-pandemic year) also had PE recorded more than 5 years previously (Supplementary eTable 3). In sensitivity analysis, we applied a consistent five-year look-back period for all patients, rather than a minimum of 5 years. For comparison, we also report episode-based counts i.e. the number of finished consultant episodes in which a relevant ICD-10 diagnosis code appeared; these are the counts published routinely by NHS England as aggregated statistics and are subject to over-estimation of events due to multiple finished consultant episodes occurring during the same admission.^23^ For both outcome measures, COVID-related PE and non–COVID-related PE were defined, respectively, by the presence or absence of the diagnosis code for COVID-19 (ICD10 U07.1) in the same hospital admission; U07.2 was added to the COVID-19 definition in sensitivity analysis.

### Statistical methods

Monthly population-based rates were calculated and expressed per 100,000 population, using mid-year population estimates obtained from the ONS as denominators.^24^ This was done for England overall and, to evaluate the temporal patterns by different patient characteristics, separately by broad age group, by sex, by region of England, and by Index of Multiple Deprivation (grouped into quintiles). Age-standardisation was conducted throughout, using the European Standard Population 2013. Crude monthly PE rates and raw counts were also calculated and reported.

Monthly rates before and after the onset of the COVID-19 pandemic (March 2020) were compared using interrupted time series analysis, and benchmarked against the pandemic “waves” as defined by the UK Office for National Statistics (ONS), the first wave spanning March to May 2020, the second from September 2020 to April 2021.^25^ To determine the significance of any temporal change in the monthly rates after February 2020, a quasi-Poisson regression model accounting for over-dispersion was fitted through the monthly counts, with the corresponding population denominators as an offset. Seasonality was captured with one annual Fourier pair (sine and cosine terms, 12-month period).^26^ Heteroskedasticity-and-autocorrelation-consistent 95% confidence intervals were derived with the Newey–West estimator (lag = 12 months, pre-whitened), thereby accommodating residual serial correlation.^26^ The model adjusted for age (5-year age groups) and sex, with month as a continuous variable to denote the underlying long-term temporal trend, and with each month from March 2020 onwards as a categorical variable, to obtain incidence rate ratios (IRRs) with 95% confidence intervals.

To examine the incidence of COVID-related PE and non–COVID-related PE by demographic subgroup (age group, sex, region, IMD quintile), incidence rate ratios were calculated using an appropriate reference category for each characteristic, adjusting for the others. First-time rates for COVID-19 hospitalisation as a primary diagnosis were also reported for reference, with overall trends and relative rates by demographic subgroup calculated in the same way. Because sex, region and IMD contained a relatively small number of missing values (Table 1), we performed complete-case analysis for each corresponding subgroup analysis.

**Table 1:** Distribution of patient characteristics for PE without co-existing COVID-19 and PE with co-existing COVID-19.

	PE without co-existing COVID-19			PE with co-existing COVID-19		
	Count (%)	Incidence rate	Adjusted rate ratio	Count (%)	Incidence rate	Adjusted rate ratio
**Age group**						
0–29	22,369 (3.1)	3.17 (3.08–3.26)	0.03 (0.03–0.03)	672 (2.1)	0.35 (0.22–0.47)	0.03 (0.02–0.03)
30–54	127,971 (17.8)	17.53 (17.09–17.98)	0.20 (0.20–0.20)	6032 (19.3)	2.67 (1.60–3.75)	0.23 (0.21–0.25)
55–64	116,450 (16.2)	18.28 (17.85–18.71)	0.53 (0.52–0.53)	5735 (18.3)	2.70 (1.51–3.89)	0.69 (0.64–0.75)
65–74^a^	173,113 (24.1)	33.86 (33.30–34.41)	1	6736 (21.5)	3.89 (2.57–5.20)	1
75–84	181,809 (25.3)	58.11 (57.00–59.22)	1.69 (1.68–1.70)	7358 (23.5)	7.09 (5.29–8.90)	1.82 (1.69–1.97)
85+	97,067 (13.5)	37.39 (36.58–38.20)	2.30 (2.29–2.32)	4797 (15.3)	5.67 (4.34–7.01)	3.25 (2.98–3.54)
**Sex**						
Male	343,005 (47.7)	6.39 (6.24–6.54)	1.09 (1.08–1.09)	18,108 (57.8)	1.03 (0.66–1.40)	1.66 (1.58–1.75)
Female^a^	375,675 (52.3)	6.83 (6.67–6.98)	1	13,218 (42.2)	0.73 (0.52–0.95)	1
Missing	99 (0.0)			4 (0.0)		
**Region**						
North East	40,272 (5.6)	7.56 (7.35–7.76)	1.25 (1.24–1.27)	1465 (4.7)	0.95 (0.70–1.20)	1.28 (1.11–1.49)
North West	100,745 (14.0)	6.91 (6.72–7.09)	1.19 (1.18–1.20)	4801 (15.3)	1.03 (0.72–1.34)	1.49 (1.32–1.67)
Yorkshire and Humber	74,101 (10.3)	6.77 (6.61–6.94)	1.17 (1.16–1.18)	3192 (10.2)	0.94 (0.70–1.18)	1.35 (1.19–1.54)
East Midlands^a^	56,076 (7.8)	5.88 (5.73–6.03)	1	2067 (6.6)	0.69 (0.51–0.87)	1
West Midlands	69,231 (9.6)	6.45 (6.29–6.62)	1.13 (1.12–1.14)	3161 (10.1)	0.96 (0.67–1.26)	1.44 (1.27–1.64)
East of England	81,579 (11.3)	6.62 (6.45–6.80)	1.10 (1.09–1.11)	2898 (9.2)	0.77 (0.53–1.00)	1.09 (0.96–1.24)
London	82,625 (11.5)	4.72 (4.61–4.83)	1.06 (1.05–1.07)	6111 (19.5)	1.07 (0.53–1.62)	2.08 (1.85–2.33)
South East	113,864 (15.8)	6.83 (6.65–7.02)	1.14 (1.12–1.15)	4462 (14.2)	0.86 (0.56–1.16)	1.21 (1.07–1.36)
South West	78,883 (11.0)	7.11 (6.96–7.25)	1.09 (1.08–1.10)	2465 (7.9)	0.72 (0.54–0.90)	0.95 (0.83–1.09)
Missing	21,403 (3.0)			708 (2.3)		
**IMD quintile**						
1 (Most deprived)	148,359 (20.6)	6.73 (6.59–6.88)	1.43 (1.42–1.45)	7704 (24.6)	1.07 (0.72–1.42)	1.90 (1.83–1.97)
2	141,560 (19.7)	6.35 (6.21–6.49)	1.24 (1.23–1.25)	6850 (21.9)	0.95 (0.61–1.29)	1.56 (1.50–1.62)
3	143,523 (20.0)	6.53 (6.38–6.68)	1.13 (1.12–1.14)	6039 (19.3)	0.86 (0.59–1.14)	1.25 (1.21–1.30)
4	142,488 (19.8)	6.63 (6.46–6.80)	1.08 (1.07–1.09)	5560 (17.7)	0.82 (0.56–1.07)	1.10 (1.06–1.14)
5 (Least deprived)^a^	132,190 (18.4)	6.33 (6.16–6.50)	1	4833 (15.4)	0.73 (0.50–0.96)	1
Missing	10,659 (1.5)			344 (1.1)		
**Month of admission**						
January	75,572	7.56 (6.93–8.20)	1.20 (1.19–1.22)	6149	2.33 (−0.70 to 5.36)	5.10 (4.79–5.44)
February	66,865	6.72 (6.08–7.35)	1.07 (1.06–1.08)	2696	1.02 (0.04–2.01)	2.31 (2.15–2.48)
March	67,643	6.74 (6.06–7.42)	1.07 (1.06–1.09)	2171	0.82 (0.44–1.21)	1.68 (1.56–1.80)
April	65,965	6.26 (5.65–6.87)	1.00 (0.99–1.01)	2573	0.98 (0.12–1.83)	2.06 (1.92–2.21)
May	68,184	6.49 (5.84–7.14)	1.03 (1.02–1.05)	1150	0.44 (0.22–0.65)	0.98 (0.90–1.07)
June^a^	66,323	6.29 (5.71–6.87)	1	1181	0.37 (0.11–0.63)	1
July	69,943	6.65 (6.04–7.26)	1.06 (1.04–1.07)	1823	0.58 (0.12–1.03)	1.57 (1.46–1.69)
August	71,338	6.79 (6.12–7.45)	1.08 (1.07–1.09)	1698	0.64 (0.14–1.15)	1.40 (1.30–1.51)
September	71,627	6.82 (6.19–7.45)	1.08 (1.07–1.10)	2066	0.65 (0.24–1.07)	1.50 (1.40–1.61)
October	75,118	7.12 (6.44–7.79)	1.13 (1.12–1.15)	2744	1.04 (0.57–1.51)	1.99 (1.86–2.13)
November	73,778	7.02 (6.35–7.69)	1.12 (1.11–1.13)	3101	0.98 (0.11–1.84)	2.36 (2.21–2.53)
December	72,203	6.87 (6.27–7.48)	1.09 (1.08–1.11)	3974	1.25 (0.14–2.37)	2.93 (2.74–3.12)

a Reference group; Incidence rates are calculated as the monthly average number of events (first-time PE-related hospital diagnosis) per 100,000 population. The adjusted rate ratios compare the average monthly population-based rate in each subgroup with that of the reference group, using data from 2008 to 2024 for PE without co-existing COVID-19, and 2020 to 2024 for PE with co-existing COVID-19. Each characteristic is adjusted for the others.

To explore trends in PE recorded as the primary diagnosis, we separately analysed first-time hospital-recorded PE restricted to the primary position. Additionally, to corroborate our results using a data-driven time series method, we analysed the data using the joinpoint method.^27^ All analyses were performed using R version 4.3.0.

### Role of the funding source

The funders of this study had no role in study design, data collection, data analysis, data interpretation, writing the report or decision to submit.

## Results

From April 2008 to December 2024, there were 750,109 first-time diagnoses of PE (mean age 67.3 years [SD 16.7]; 47.7% male) and 1,709,672 PE-related finished consultant episodes. The selection of patients into the study is described in Supplementary eFig. 1, and the characteristics of patients with COVID-related PE and non–COVID-related PE are described in Table 1.

**Fig. 1:**
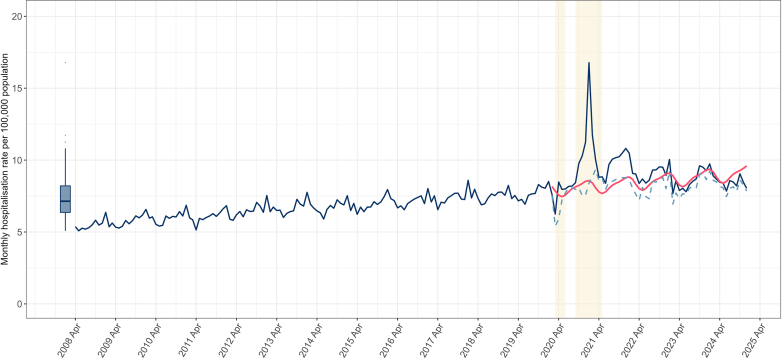
Age-standardised first-time PE incidence rates with and without co-existing COVID-19 in England, 2008–2024. Numerators represent the number of first-time hospital admissions with pulmonary embolism (PE) per patient, recorded in any diagnostic position. Denominators are the mid-year population estimates from the Office for National Statistics. Rates are age-standardised to the 2013 European Standard Population. Box plots show the historical average age-standardised PE rate from April 2008 to December 2024 (median and interquartile range). The shaded area marks the first and second waves of the COVID-19 pandemic in England. Red line – adjusted counterfactual (expected trend based on the pre-pandemic period). Blue line – observed age-standardised rate. Blue dots – observed age-standardised rate without co-existing COVID-19.

**Fig. 2:**
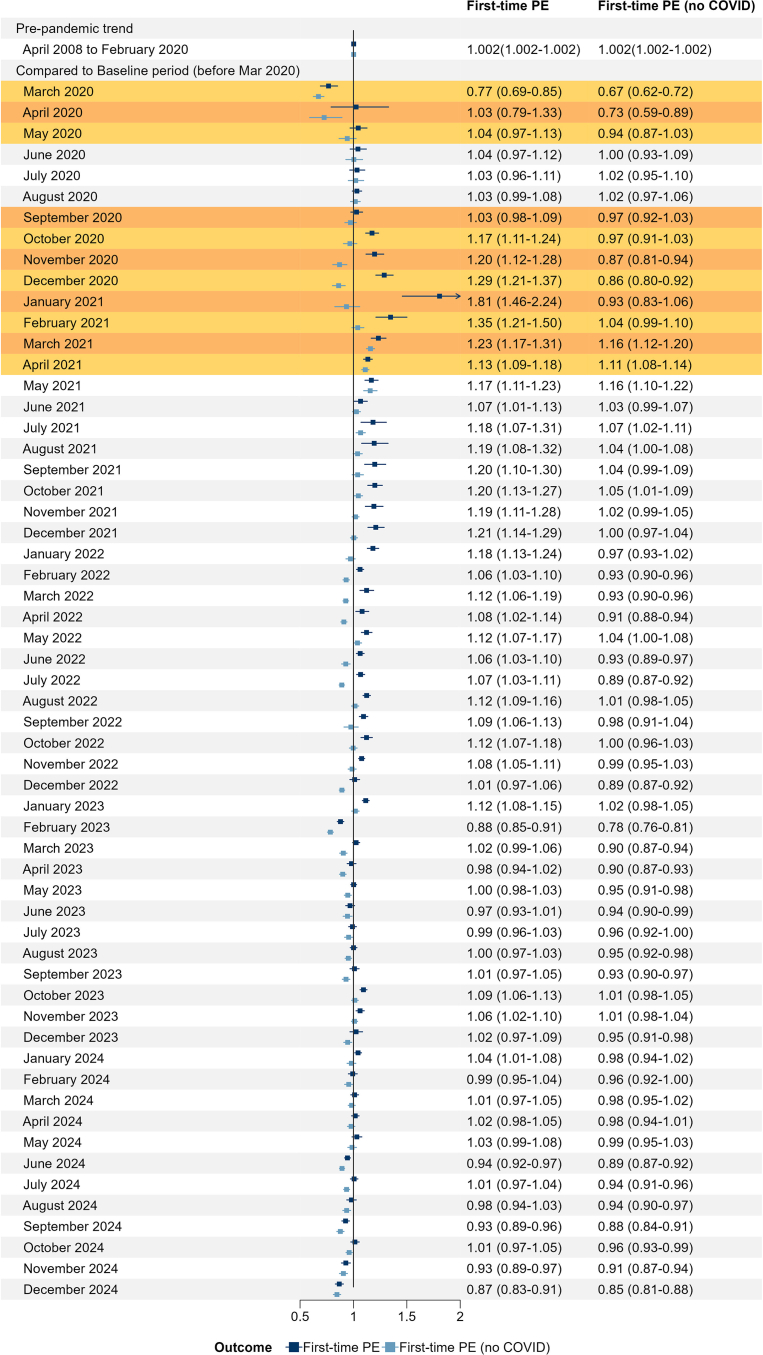
Effect of the COVID-19 pandemic on first-time PE incidence rates, with and without co-existing COVID-19, comparing post-pandemic months (March 2020 to December 2024) with pre-pandemic trend (April 2008 to February 2020). The model is adjusted for age (5-year age groups) and sex, with month treated as a continuous variable and each month from March 2020 onwards treated as a categorical variable. The IRR for the pre-pandemic trend represents the modelled increase per month observed during the pre-pandemic period. It is against this baseline trend, and its hypothetical continuation, that the months from March 2020 onwards are compared. For example, the IRR of 1.81 in January 2021 means the observed rate that month was 81% higher than would have been expected had the pre-pandemic trend continued. The orange shaded area indicates the first and second waves of the coronavirus pandemic in England.

Age-standardised temporal trends in first-time PE diagnoses are shown in Fig. 1. There was a long-term increase in age-standardised rates from 5.4 per 100,000 population in April 2008 to 8.5 per 100,000 in January 2020. Seasonal variation was evident throughout the pre-pandemic period (Fig. 1); for instance, rates in January were, on average, 20% higher than those in June (Table 1). Rates dipped overall in March 2020 at the onset of the pandemic, then rose sharply to 16.8 per 100,000 in January 2021. Comparing observed levels of first-time PE diagnoses in January 2021 with expected levels based on the pre-pandemic trend (i.e. the counterfactual), the IRR was 1.81 (95% CI, 1.46–2.24); Fig. 2. The January 2021 spike was largely accounted for by admissions with co-existing COVID-19 infection: COVID-related PE accounted for 50% of first-time PEs that month (8.4 per 100,000 population). Rates returned to 2019 levels (7.7 per 100,000) by December 2022 (Fig. 1; Fig. 2). Since then, rates plateaued and largely remained at pre-pandemic levels until mid-2024, whereas from June 2024 onwards the IRRs have mostly remained below 1, particularly in the latter months of 2024: the relative decline in December 2024 compared with pre-pandemic trends was 0.87 (95% CI, 0.83–0.91).

Age-standardised temporal trends by demographic subgroup are illustrated in Supplementary eFigs. 2–5. Temporal trends were broadly consistent across age groups; however, the overall dip observed during the first wave of the pandemic was driven only by older age groups, as younger age groups (<65) experienced significant spikes at this time; all age groups experienced very substantial increases during the second wave (Supplementary eFig. 2). Age-standardised temporal trends were broadly similar for both sexes, except during the first wave when women experienced a reduction while men experienced a significant increase; both men and women experienced substantial increases during the second wave (Supplementary eFig. 3). The adjusted male:female ratio for COVID-related PE (1.66 (95% CI, 1.58–1.75)) was higher than that for non-COVID related PE (1.09 (95% CI, 1.08–1.09), Table 1). Regionally, London experienced the greatest spikes in first-time PE diagnoses during the pandemic, with rates of 14.3 per 100,000 in April 2020 and 29.1 per 100,000 in January 2021, whereas the rest of England averaged 7.5 and 14.6 per 100,000 population, respectively (Supplementary eFig. 4). Variation by IMD was evident throughout (Supplementary eFig. 5): the most deprived quintile had the highest rates of non-COVID-19-related PE relative to the least deprived quintile (IRR: 1.43 (95% CI, 1.42–1.45)) and even higher relative rates of COVID-19 related PE (1.90 (95% CI, 1.83–1.97)) (Table 1), mirroring the sharp deprivation gradient observed for COVID-19 hospitalisation itself as a primary diagnosis (Supplementary eTable 1).

Episode-based PE rates were higher overall than first-time PE diagnosis rates throughout and the extent of the increase in episode-based rates over the long term was considerably greater than for first-time PE diagnoses (Supplementary eFig. 6 and eTable 2). The crude (unstandardised) and standardised rates were very similar, with closely aligned temporal trends (Supplementary eFig. 7 and eTable 2).

Findings did not materially change when applying a fixed five-year look-back (Supplementary eFigs. 8 and 9). Defining COVID-19 using either ICD-10 U07.1 or U07.2, instead of U07.1 alone, boosted COVID-related PE numbers by about 6% (33,298 vs 31,330), albeit potentially at the expense of case ascertainment specificity. In any case, the inclusion of U07.2 had no material effect on the trends (Supplementary eFig. 10). The majority of first-time hospital-recorded PEs were coded as the primary diagnosis (58.8%, n = 441,370). When restricting the case definition to PEs recorded as the primary diagnosis only, the pre-pandemic trend increased similarly to the main analysis (Supplementary eFigs. 11 and 12); however, the spike around the period of January 2021 was not apparent when we restricted the case definition to primary-diagnosis PEs. Very few COVID-related PEs remained, as COVID-19 itself was coded in the primary position in most such admissions.

Joinpoint analysis corroborated the findings of the main analysis (Supplementary eFig. 16), showing a consistent upward trend during the pre-pandemic period, a spike during the pandemic, followed by a decline in the post-pandemic period.

## Discussion

Following an upward trend from 2008 through to 2019, the overall incidence of hospital-recorded PE presentations declined between March 2020 and May 2020, which coincided with the first wave of the COVID-19 pandemic, and increased substantially in the period coinciding with the second wave (January 2021). The increase during the second wave largely reflected presentations with co-existing COVID-19 infection. Analysis by demographic subgroup revealed notable heterogeneity in trends during the COVID-19 period, with men, the more deprived, and those living in London being disproportionately affected by COVID-related PE. Rates have come down since the pandemic heights, returning to pre-pandemic levels by early 2023, and may be continuing to decline, subject to further updates.

Understanding how hospital-recorded PE incidence has changed during and since the months of the COVID-19 pandemic (2020–2021) is essential to resource allocation, pandemic preparedness, health system recovery, and benchmarking, but has not been reported in detail.^2^^,^^7^^,^^8^^,^^10^^,^^11^ A previous study, published during the early phase of the pandemic, reported an increased incidence of hospital-recorded PE per 100,000 admissions in England during the first wave (up to July 2020), largely driven by PE presentations with a co-existing COVID-19 diagnosis.^8^ However, this study did not cover the second wave, and the rates per 100,000 admissions are susceptible to fluctuations in the denominator, limiting their usefulness for understanding population-based trends in PE incidence.^8^ In contrast, a more recent study reported no significant change in population-based PE rates during the pandemic (2020–2021), but was restricted to analysis of aggregated hospital episodes at the annual level, therefore was unable to detect short-term monthly variation in incidence.^2^ Both prior studies were restricted to hospitalisations where PE was the primary diagnosis, meaning neither study captured cases where PE was recorded secondary to other causes, notably COVID-19. We show for the first time, using multiple-cause coded person-based national hospital data, that age-standardised and age-specific population-based rates of first-time PE diagnoses increased substantially in England during the second wave of the pandemic among those with a co-existing diagnosis of COVID-19 infection. COVID-19 infection is known to increase the risk of developing PE.^28^ Previous studies have found thrombosis rates to be higher than expected in COVID-19 compared with influenza.^29^, ^30^, ^31^

Hospitalisation with acute COVID-19 infection is known to be associated with increased risk of developing PE.^8^ Previous studies have found thrombosis rates to be higher than expected in COVID-19 compared with influenza.^29^, ^30^, ^31^ Therefore, it is perhaps surprising that an overall decline in PE admissions was observed during the first wave. Potential explanations include a reduction in the use of imaging techniques in hospital to diagnose PE,^32^ and changes in healthcare-seeking behaviour among the public for fear of contracting COVID-19 in hospital.^13^ Findings may therefore not reflect the true overall burden of PE incidence during the first wave. The heterogeneity by region, with rates spiking in London during the first wave but decreasing in other regions, potentially reflects the weight of these competing drivers alongside COVID-19 as a risk factor for PE. By the second wave, overall use of imaging techniques in England recovered to pre-pandemic levels,^33^ and it is likely that healthcare-seeking behaviour among PE patients increased following a public awareness campaign by the UK government encouraging patients to seek healthcare when needed.^34^ It is also conceivable that thresholds for CTPA scanning were lowered in people with COVID-19 due to increased awareness of high thrombotic risk, potentially contributing to increased detection of PE.

The epidemiological profile of COVID-related PE likely represents a skewing of the usual epidemiological profile of non-COVID related PE toward the epidemiological profile of COVID-19 (Supplementary eTable 1). For example, the increase in risk associated with social deprivation was steeper for COVID-related PE than for non-COVID PE, and mirrored the sharp deprivation gradient observed for COVID-19 hospitalisation rates. This contrasts with a previous study, which found that deprivation-level differences in cardiovascular hospitalisations were not exacerbated by the pandemic, but was based on cardiovascular conditions that were recorded as the primary diagnosis only and therefore did not account for cardiovascular events that were secondary to COVID-19. The adjusted male:female ratio of COVID-related PE (1.66 (95% CI, 1.58–1.75)) exceeded both that of non-COVID PE (1.09 (95% CI, 1.08–1.09)) and COVID-19 itself (1.33 (95% CI, 1.32–1.34)). This could be due to an interaction between COVID-19 and risk factors for COVID-related PE that are more common in men, or it could be that men are more susceptible to the pathophysiological pathways that underpin COVID-related PE,^8^^,^^28^ but this requires further investigation.

The findings of a long-term upward trend in presentations to hospital with PE in this study prior to the pandemic are broadly consistent with findings from other studies that have used hospital data to report an increase in PE diagnoses in England,^2^^,^^35^ as well as in other countries.^36^, ^37^, ^38^, ^39^, ^40^ Our findings on seasonal variation strengthen inconclusive European evidence suggesting a winter peak in non-COVID-related PE, which may driven by other seasonal respiratory infections.^41^^,^^42^ Our comparison of first-time diagnosis rates with ‘episode-based’ activity demonstrates that this observed long-term upward trend is not simply an artefact of increased repeat recording of the same clinical events. The improved detection of PE with the increased use and performance of computed tomographic pulmonary angiography (CTPA), which is highly sensitive in detecting sub-segmental PEs, has likely contributed to this long-term upward trend.^2^^,^^43^^,^^44^ An increased prevalence of comorbidities that predispose to PE, such as obesity and cancer,^45^^,^^46^ may have also contributed to these trends, along with a gradual increase over time in surgical procedures.^47^, ^48^, ^49^

In recent years, monthly rates of first-time PE admissions have come down from the heights reached during the COVID-19 pandemic. Vaccination against COVID-19 is likely to have contributed to the observed reduction in COVID-related PE rates through reduced instances of COVID-19 infection, particularly severe disease.^50^^,^^51^ It is too early to determine whether the recent downward trend may begin to reverse some of the consistent upward trend observed over the decade before the pandemic. In 2020, guidelines from the National Institute for Health and Care Excellence (NICE) guidelines in England recommended that clinicians^16^^,^^17^ but pandemic-related disruption and reorganisation of health services and the NICE guidelines may have further catalysed the shift. A shift from inpatient to outpatient care for low-risk patients effectively managed out of hospital would impact inpatient-recorded rates. However, we are unable to analyse the distribution of PE care across different management pathways without direct reference to data on other care pathways. Data on PE events treated out of hospital may be recorded at the primary care level, particularly if anticoagulants are prescribed, but were not available for this study. Ongoing monitoring of PE incidence by healthcare setting is important to undertake while clinical practice and policy on PE management pathways evolve, since it helps to support care planning; it also informs data-enabled clinical trials where PE is an outcome as well as the design of observational studies. The ability to use routine data to track patients from the at-risk stage (such as post-surgery or after a deep vein thrombosis diagnosis) where prophylactic antithrombotic therapy may be prescribed, through PE diagnosis—including detailed information on management pathways—and subsequent post-PE outcomes, needs to improve. More comprehensive data at the regional level in England are becoming available to explore.^52^^,^^53^

These findings have important translational implications for current and future clinical practice. The observed spike in pulmonary embolism (PE) incidence during the second wave of COVID-19, particularly among subgroups already at heightened risk of severe disease, emphasises the need for vigilance around thrombotic complications in respiratory infections and in vulnerable populations. The fact that PE incidence remains shaped by changing diagnostic and care pathways highlights the need for ongoing monitoring across inpatient, ambulatory, and primary care settings. Strengthening linkage between routine datasets, including primary care prescribing and outpatient records, will be critical for understanding the full scope of PE burden, ensuring equitable access to appropriate care, and supporting the evaluation of prevention strategies. These findings also underline the value of real-time, population-based surveillance systems that can capture rapid shifts in disease patterns, which are essential not only for pandemic preparedness but for improving responsiveness in routine care.

We were able to report contemporary and historical incidence rates of hospital-recorded PE with close to complete coverage for England. Record-linkage of contiguous hospital episodes enabled the identification of first-time PE admission rates, which could be compared with ‘episode-based’ PE rates that are subject to over-counting. Internal linkage of multiple-cause coded HES records also enabled exploration of the contribution of COVID-19 as a co-existing diagnosis, and reporting of rates of both COVID-19-related and non-COVID-19-related PE events, which appear to have different phenotypes.^28^ Our method using record linkage comprehensively captures all first-time hospital-recorded PE, whether recorded secondary to COVID-19 or another condition, without over-counting.

Precision of analysis by month enabled assessment of PE hospitalisation rates during the pandemic in England, and in light of other contemporary influences, including systemic disruption to the health service since the COVID-19 pandemic,^15^ and evolving practices for PE diagnosis, prevention and management.^16^^,^^17^ However, we were unable to establish these factors as causes of the observed trends. Future updates to the analyses in this study will continue to track hospital-recorded PE rates at the national level through an online tool to inform emergency care planning, public health messaging, and policy guidance relating to the diagnosis and management of PE.

The study captured all PEs diagnosed in hospital in the HES-APC dataset analysed, which is the standard pathway for PE diagnosis in England. However, data sources on other care pathways were not available. PEs diagnosed and treated without admission to hospital may be captured by linkage to primary care data, especially where anticoagulants are prescribed, but primary care data was not available for this study. PEs diagnosed and treated under the NHS’s ‘same-day emergency care’ (SDEC) strategy, first implemented in 2019, may not have been consistently captured in the HES-APC data, creating uncertainty about their effect on the trends reported here.^54^

All analyses were reliant on the accuracy of diagnosis coding of both PE and COVID-19. The ICD-10 coding for both of these conditions has been shown to be reliable.^18^^,^^19^^,^^55^ The accuracy of the population-based rates is also dependent on the accuracy of the mid-year population estimates obtained from the ONS, which were applied to each month of the respective calendar year; while small within-year variations may occur, these are unlikely to be larger than the typical year-to-year changes in mid-year population estimates, which on average varied by only 0.69% from one year to the next.

Data from other countries that include the pandemic and post-pandemic periods are needed to clarify the generalisability of our findings and the impact on healthcare systems in other regions.

This study shows that following an upward trend from 2008 through to 2019, the overall incidence of hospital-recorded PE spiked substantially overall in the period coinciding with the second wave (January 2021), before returning to 2019 levels by early 2023. There was heterogeneity in the pandemic trends by demographic subgroup that reflected the differential impact of COVID-19. The preceding long-term increase in PE hospitalisation shows that a continued emphasis on improved prevention of PE is needed, including through prevention and management of underlying risk factors, appropriate timing of hospital discharge, and the use of effective antithrombotic therapy. The plateauing of rates since 2023, which represents a break from the upward trend observed over the prior 15 years, warrants further investigation. Continued surveillance of PE incidence and outcomes with reference to detailed patient-level data on preventive, diagnostic, and treatment pathways will be essential to assess the effectiveness of ongoing prevention and management strategies. Future updates to the analyses in this study will continue to track inpatient-recorded PE rates at the national level to support emergency care planning and to inform and evaluate evolving strategies for PE diagnosis, prevention, and management.

## Contributors

All authors included on the paper fulfil the criteria of authorship. All authors (X. Zhong, J. Webster, E. Morris, E. Oguzman, S. Shapiro, S. Shepperd, and R. Goldacre) contributed to study concept, design and review of the manuscript. X. Zhong, J. Webster, and R. Goldacre devised the statistical strategy. E. Oguzman developed the online tool. J. Webster conducted the literature search. X. Zhong conducted the data analysis and generated the figures. X. Zhong and R. Goldacre had full access to all the data in the study and take responsibility for the integrity of the data and the accuracy of the data analysis. R. Goldacre verified the data. X. Zhong, J. Webster, and R. Goldacre drafted the manuscript. R. Goldacre had final responsibility for decision to submit for publication.

## Data sharing statement

X. Zhong and R. Goldacre had full access to all the data in the study and take responsibility for the integrity of the data and the accuracy of the data analysis. All data used in this study are available through application to NHS England at https://digital.nhs.uk/.

## Declaration of interests

None to declare.

## Appendix A Supplementary data

Supplementary eFigs. 1–16 and eTables 1–3

Supplementary material online tool

Supplementary material STROBE
