# Supplementary material for: Temporal trends in hospital-recorded pulmonary embolism in England before, during and after the COVID-19 pandemic (2008–2024): a population-based observational study
Source: Lancet Reg Health Eur. 2025 Sep 2;58:101433. doi: 10.1016/j.lanepe.2025.101433 (PMC12444491; doi:10.1016/j.lanepe.2025.101433)
Supplement: Supplementary eFigs. 1–16 and eTables 1–3 [file mmc1.pdf]

supplementary material

eText 1 Supplementary methods 2

eTable 1 Distribution of patient characteristics for COVID-related pulmonary embolism, non-COVID-related pulmonary embolism, and COVID-19 separately in England, April 2008 to December 2024. 3

eTable 2 Underlying counts and rates (age-standardised and crude) for episode-based and first-time PE-related hospital diagnoses over time in England, April 2008–December 2024. 5

eTable 3 Prior Pulmonary Embolism History for Patients Admitted in 2019, Demonstrating the Adequacy of a Five-Year Look-back Window (n = 56 143) 14

eFigure 1 Flow diagram of individuals included in the analysis with first-time hospital-recorded PE from April 2008 through December 2024. 15

eFigure 2 First-time hospital-recorded PE rates by age group (age-standardised), April 2008 to December 2024 16

eFigure 3 First-time hospital-recorded PE rates by sex (age-standardised), April 2008 to December 2024 17

eFigure 4 First-time hospital-recorded PE rates by the 9 regions (age-standardised), April 2008 to December 2024 18

eFigure 5 First-time hospital-recorded PE rates by IMD quintile (age-standardised), April 2008 to December 2024 19

eFigure 6 First-time, episode-based hospital-recorded PE rates (age-standardised), April 2008 to December 2024 20

eFigure 7 First-time, episode-based hospital-recorded PE rates (crude), April 2008 to December 2024 21

eFigure 8 Effect of the COVID-19 pandemic on first-time PE incidence rates, with and without co-existing COVID-19, comparing post-pandemic months (March 2020 to December 2024) with pre-pandemic trend (April 2008 to February 2020); cohort with a consistent 5-year look-back history 22

eFigure 9 First-time hospital-recorded PE rates: comparison of cohorts with (i) a minimum 5-year look-back history (main study population) and (ii) a consistent 5-year look-back history, April 2008–December 2024 23

eFigure 10 Effect of the COVID-19 pandemic on first-time PE incidence rates, with and without co-existing COVID-19, comparing post-pandemic months (March 2020 to December 2024) with pre-pandemic trend (April 2008 to February 2020); COVID diagnosis using ICD-10 U07.1 and U07.2 24

eFigure 11 First-time hospital-recorded PE rates by diagnosis position, any position (blue) vs primary (first) position (green), April 2008 – December 2024 25

eFigure 12 Effect of the COVID-19 pandemic on first-time PE incidence rates, with and without co-existing COVID-19, comparing post-pandemic months (March 2020 to December 2024) with pre-pandemic trend (April 2008 to February 2020); PE in primary(first) position 26

eFigure 13 First-time hospital-recorded PE rates: 42 Integrated Care Boards (ICBs) versus all 44 ICBs, April 2008 – December 2024 27

eFigure 14 Effect of the COVID-19 pandemic on first-time PE incidence rates, with and without co-existing COVID-19, comparing post-pandemic months (March 2020 to December 2024) with pre-pandemic trend (April 2008 to February 2020); All 44 ICBs included 28

eFigure 15 First-time hospital-recorded PE rates (any position) and first-time hospital-recorded COVID-19 (primary position), April 2008 to December 2024 29

eFigure 16 Joinpoint analysis of first-time hospital-recorded PE: any-position versus primary-diagnosis-only cases/with and without co-existing COVID-19 30

eText 1 Supplementary methods

We used mid-year population estimates from the Office for National Statistics (ONS) to calculate population-based rates in England. Two sets of mid-year estimates were obtained: national rebased estimates (revised after the 2021 Census) for 2008–2023,[1] and Lower Layer Super Output Area (LSOA) estimates for 2008–2020.[2] The national rebased estimates provided denominators by age group and sex, as well as for the total population, and the 2023 estimates were also used for 2024 data.

To account for deprivation, we applied the English Indices of Deprivation (IMD), which measure relative deprivation in LSOAs.[3] The latest version (IMD 2019) is based on 2011 Census boundaries, and the 2020 LSOA dataset is the last to use these boundaries.[1] We first mapped each IMD quintile to LSOA estimates from 2008 to 2020: estimates for 2008–2016 were matched to IMD 2015, while those for 2017–2020 were matched to IMD 2019. We then applied the observed annual IMD distribution to the national rebased estimates by age and sex for 2008–2023, using the 2020 distribution for 2021–2023. The 2023 estimates were again used for 2024 denominators. The same approach was used for Integrated Care Boards (ICBs), ICB levels were derived using the ONS correspondence table.[4]

Frimley Health NHS Foundation Trust (RDU) was unable to submit complete HES records for June 2022–March 2023 due to missing data reported by NHS England.[5] Similarly, Shrewsbury and Telford Hospital NHS Trust (RXW) could not upload records from early July 2024.[6] These gaps consequently affected the national counts. To avoid under-ascertainment, we excluded all records from NHS Shropshire, Telford and Wrekin Integrated Care Board and NHS Frimley Integrated Care Board for the entire study period (April 2008 – December 2024). The resident populations of these two ICBs were removed from the denominators for every month accordingly (eFigure 1). To evaluate whether removing the two affected ICBs alters regional and national trend estimates, we repeated the trends plot and the ITS model using the full England-population denominator.

References

1. Office for National Statistics. *Population estimates - local authority based by single year of age*. 2023. Accessed May 22, 2025. https://www.nomisweb.co.uk/datasets/pestsyoala

2. Office for national Statistics. *Lower layer Super Output Area population estimates (supporting information).* 2024. Accessed May 22, 2025. https://www.ons.gov.uk/peoplepopulationandcommunity/populationandmigration/populationestimates/datasets/lowersuperoutputareamidyearpopulationestimates

3. Ministry of Housing CLGt. *English indicies of deprivation 2019*. 2019. Accessed Oct 10, 2025. https://www.gov.uk/government/statistics/english-indices-of-deprivation-2019

4. Office for National Statistics. *LSOA (2011) to Sub Integrated Care Board Locations (July 2022) Lookup in EN*. 2022. Accessed May 22, 2025. https://geoportal.statistics.gov.uk/datasets/ons::lsoa-2011-to-sub-integrated-care-board-locations-july-2022-lookup-in-en/about

5. NHS England. *Hospital Admitted Patient Care Activity, 2022-23*. 2023. Accessed 22 May, 2025. https://digital.nhs.uk/data-and-information/publications/statistical/hospital-admitted-patient-care-activity/2022-23/about-this-publication#data-quality

6. NHS England. *Hospital Admitted Patient Care Activity*. 2024. Accessed May 22, 2025. https://digital.nhs.uk/data-and-information/publications/statistical/hospital-admitted-patient-care-activity

eTable 1 Distribution of patient characteristics for COVID-related pulmonary embolism, non-COVID-related pulmonary embolism, and COVID-19 separately in England, April 2008 to December 2024.

	Non-COVID-related PE			COVID-related PE			COVID		
	Count (%)	incidence rate	Adjusted rate ratio	Count (%)	incidence rate	Adjusted rate ratio	Count (%)	incidence rate	Adjusted rate ratio
Age group									
0-29	22369 (3.1)	3.17 (3.08 to 3.26)	0.03 (0.03 to 0.03)	672 (2.1)	0.35 (0.22 to 0.47)	0.03 (0.02 to 0.03)	54439 (9.4)	27.52 (21.70 to 33.34)	0.14 (0.13 to 0.14)
30-54	127971 (17.8)	17.53 (17.09 to 17.98)	0.20 (0.20 to 0.20)	6032 (19.3)	2.67 (1.60 to 3.75)	0.23 (0.21 to 0.25)	112537 (19.4)	52.42 (34.55 to 70.29)	0.31 (0.30 to 0.31)
55-64	116450 (16.2)	18.28 (17.85 to 18.71)	0.53 (0.52 to 0.53)	5735 (18.3)	2.70 (1.51 to 3.89)	0.69 (0.64 to 0.75)	78408 (13.5)	39.33 (24.70 to 53.96)	0.63 (0.63 to 0.64)
65-74*	173113 (24.1)	33.86 (33.30 to 34.41)	1	6736 (21.5)	3.89 (2.57 to 5.20)	1	96168 (16.6)	59.26 (40.88 to 77.65)	1
75-84	181809 (25.3)	58.11 (57.00 to 59.22)	1.69 (1.68 to 1.70)	7358 (23.5)	7.09 (5.29 to 8.90)	1.82 (1.69 to 1.97)	128463 (22.2)	132.10 (95.69 to 168.51)	2.16 (2.14 to 2.18)
85+	97067 (13.5)	37.39 (36.58 to 38.20)	2.30 (2.29 to 2.32)	4797 (15.3)	5.67 (4.34 to 7.01)	3.25 (2.98 to 3.54)	109848 (18.9)	134.59 (98.25 to 170.94)	4.82 (4.78 to 4.87)
Sex				`					
Male	343005 (47.7)	6.39 (6.24 to 6.54)	1.09 (1.08 to 1.09)	18108 (57.8)	1.03 (0.66 to 1.40)	1.66 (1.58 to 1.75)	302717 (52.2)	18.67 (13.08 to 24.27)	1.33 (1.32 to 1.34)
Female*	375675 (52.3)	6.83 (6.67 to 6.98)	1	13218 (42.2)	0.73 (0.52 to 0.95)	1	276233 (47.6)	16.67 (12.21 to 21.14)	1
Missing	99 (0.0)			4 (0.0)			913 (0.2)		
Region									
A = North East	40272 (5.6)	7.56 (7.35 to 7.76)	1.25 (1.24 to 1.27)	1465 (4.7)	0.95 (0.70 to 1.20)	1.28 (1.11 to 1.49)	34257 (5.9)	21.54 (16.44 to 26.65)	1.42 (1.40 to 1.44)
B = North West	100745 (14.0)	6.91 (6.72 to 7.09)	1.19 (1.18 to 1.20)	4801 (15.3)	1.03 (0.72 to 1.34)	1.49 (1.32 to 1.67)	86777 (15.0)	19.89 (14.25 to 25.53)	1.34 (1.33 to 1.36)
D = Yorkshire and Humber	74101 (10.3)	6.77 (6.61 to 6.94)	1.17 (1.16 to 1.18)	3192 (10.2)	0.94 (0.70 to 1.18)	1.35 (1.19 to 1.54)	61528 (10.6)	18.78 (14.30 to 23.26)	1.27 (1.26 to 1.29)
E = East Midlands*	56076 (7.8)	5.88 (5.73 to 6.03)	1	2067 (6.6)	0.69 (0.51 to 0.87)	1	42711 (7.4)	14.81 (10.97 to 18.64)	1
F = West Midlands	69231 (9.6)	6.45 (6.29 to 6.62)	1.13 (1.12 to 1.14)	3161 (10.1)	0.96 (0.67 to 1.26)	1.44 (1.27 to 1.64)	63353 (10.9)	19.59 (13.87 to 25.32)	1.34 (1.33 to 1.36)
G = East of England	81579 (11.3)	6.62 (6.45 to 6.80)	1.10 (1.09 to 1.11)	2898 (9.2)	0.77 (0.53 to 1.00)	1.09 (0.96 to 1.24)	56623 (9.8)	15.22 (10.85 to 19.59)	1.00 (0.99 to 1.01)
H = London	82625 (11.5)	4.72 (4.61 to 4.83)	1.06 (1.05 to 1.07)	6111 (19.5)	1.07 (0.53 to 1.62)	2.08 (1.85 to 2.33)	96993 (16.7)	18.20 (10.99 to 25.41)	1.56 (1.54 to 1.58)
J = South East	113864 (15.8)	6.83 (6.65 to 7.02)	1.14 (1.12 to 1.15)	4462 (14.2)	0.86 (0.56 to 1.16)	1.21 (1.07 to 1.36)	76931 (13.3)	15.31 (10.27 to 20.36)	1.00 (0.98 to 1.01)
K = South West	78883 (11.0)	7.11 (6.96 to 7.25)	1.09 (1.08 to 1.10)	2465 (7.9)	0.72 (0.54 to 0.90)	0.95 (0.83 to 1.09)	47879 (8.3)	14.24 (10.92 to 17.56)	0.86 (0.85 to 0.88)
Missing	21403 (3.0)			708 (2.3)			12811 (2.2)		

IMD quintile									
1 (Most deprived)	148359 (20.6)	6.73 (6.59 to 6.88)	1.43 (1.42 to 1.45)	7704 (24.6)	1.07 (0.72 to 1.42)	1.90 (1.83 to 1.97)	147484 (25.4)	22.18 (15.75 to 28.62)	2.04 (2.02 to 2.06)
2	141560 (19.7)	6.35 (6.21 to 6.49)	1.24 (1.23 to 1.25)	6850 (21.9)	0.95 (0.61 to 1.29)	1.56 (1.50 to 1.62)	124931 (21.5)	18.50 (12.90 to 24.10)	1.58 (1.57 to 1.60)
3	143523 (20.0)	6.53 (6.38 to 6.68)	1.13 (1.12 to 1.14)	6039 (19.3)	0.86 (0.59 to 1.14)	1.25 (1.21 to 1.30)	111316 (19.2)	16.75 (11.94 to 21.55)	1.29 (1.28 to 1.30)
4	142488 (19.8)	6.63 (6.46 to 6.80)	1.08 (1.07 to 1.09)	5560 (17.7)	0.82 (0.56 to 1.07)	1.10 (1.06 to 1.14)	102269 (17.6)	15.80 (11.48 to 20.11)	1.15 (1.14 to 1.16)
5 (Least deprived) *	132190 (18.4)	6.33 (6.16 to 6.50)	1	4833 (15.4)	0.73 (0.50 to 0.96)	1	88741 (15.3)	14.17 (10.38 to 17.96)	1
Missing	10659 (1.5)			344 (1.1)			5122 (0.9)		
Month									
January	63885 (8.9)	7.56 (6.93 to 8.20)	1.20 (1.19 to 1.22)	6149 (19.6)	2.33 (-0.70 to 5.36)	5.10 (4.79 to 5.44)	97939 (16.9)	46.38 (-6.12 to 98.87)	5.36 (5.28 to 5.44)
February	56735 (7.9)	6.72 (6.08 to 7.35)	1.07 (1.06 to 1.08)	2696 (8.6)	1.02 (0.04 to 2.01)	2.31 (2.15 to 2.48)	40787 (7.0)	15.45 (2.83 to 28.07)	1.81 (1.78 to 1.84)
March	56908 (7.9)	6.74 (6.06 to 7.42)	1.07 (1.06 to 1.09)	2171 (6.9)	0.82 (0.44 to 1.21)	1.68 (1.56 to 1.80)	55110 (9.5)	20.88 (7.04 to 34.72)	2.41 (2.37 to 2.45)
April	56206 (7.8)	6.26 (5.65 to 6.87)	1.00 (0.99 to 1.01)	2576 (8.2)	0.98 (0.12 to 1.83)	2.06 (1.92 to 2.21)	59030 (10.2)	22.36 (-1.09 to 45.82)	2.58 (2.54 to 2.62)
May	58240 (8.1)	6.49 (5.84 to 7.14)	1.03 (1.02 to 1.05)	1150 (3.7)	0.44 (0.22 to 0.65)	0.98 (0.90 to 1.07)	22039 (3.8)	8.35 (3.86 to 12.84)	0.97 (0.95 to 0.98)
June*	56430 (7.9)	6.29 (5.71 to 6.87)	1	1181 (3.8)	0.37 (0.11 to 0.63)	1	22992 (4.0)	8.71 (3.14 to 14.28)	1
July	59654 (8.3)	6.65 (6.04 to 7.26)	1.06 (1.04 to 1.07)	1823 (5.8)	0.58 (0.12 to 1.03)	1.57 (1.46 to 1.69)	39974 (6.9)	15.14 (3.41 to 26.88)	1.75 (1.72 to 1.78)
August	60914 (8.5)	6.79 (6.12 to 7.45)	1.08 (1.07 to 1.09)	1698 (5.4)	0.64 (0.14 to 1.15)	1.40 (1.30 to 1.51)	31254 (5.4)	11.84 (2.54 to 21.14)	1.37 (1.35 to 1.39)
September	61217 (8.5)	6.82 (6.19 to 7.45)	1.08 (1.07 to 1.10)	2066 (6.6)	0.65 (0.24 to 1.07)	1.50 (1.40 to 1.61)	36017 (6.2)	13.64 (7.64 to 19.65)	1.57 (1.54 to 1.59)
October	63882 (8.9)	7.12 (6.44 to 7.79)	1.13 (1.12 to 1.15)	2744 (8.8)	1.04 (0.57 to 1.51)	1.99 (1.86 to 2.13)	54100 (9.3)	20.49 (11.07 to 29.92)	2.36 (2.32 to 2.40)
November	63006 (8.8)	7.02 (6.35 to 7.69)	1.12 (1.11 to 1.13)	3101 (9.9)	0.98 (0.11 to 1.84)	2.36 (2.21 to 2.53)	50134 (8.6)	18.99 (2.28 to 35.70)	2.19 (2.16 to 2.23)
December	61702 (8.6)	6.87 (6.27 to 7.48)	1.09 (1.08 to 1.11)	3975 (12.7)	1.25 (0.14 to 2.37)	2.93 (2.74 to 3.12)	70487 (12.2)	26.70 (4.56 to 48.84)	3.08 (3.03 to 3.13)

*Reference group. Crude rates are calculated as the monthly average number of events (first-time PE-related hospital diagnosis) per 100,000 population. For all subgroups except COVID-related PE and COVID-19, the denominator is the subgroup’s average population from 2008 to 2024; for COVID-related PE and COVID-19, it is the average population from 2020 to 2024. All denominators are based on mid-year estimates from the Office for National Statistics. Rate ratios are adjusted for all variables shown in this table. COVID-19 indicates first-time COVID-19 hospitalisation, derived from a separate data extraction in which COVID-19 is the primary diagnosis of the admission.

eTable 2 Underlying counts and rates (age-standardised and crude) for episode-based and first-time PE-related hospital diagnoses over time in England, April 2008–December 2024.

month	count	age-standardised rate	crude rate	type
01/04/2008	4356	9.76	8.60	Episode-based
01/05/2008	4139	9.26	8.17	Episode-based
01/06/2008	4452	9.99	8.79	Episode-based
01/07/2008	4329	9.70	8.54	Episode-based
01/08/2008	4523	10.10	8.93	Episode-based
01/09/2008	4572	10.23	9.02	Episode-based
01/10/2008	4948	11.09	9.77	Episode-based
01/11/2008	4506	10.10	8.89	Episode-based
01/12/2008	4531	10.19	8.94	Episode-based
01/01/2009	5362	11.87	10.51	Episode-based
01/02/2009	4474	9.93	8.77	Episode-based
01/03/2009	4828	10.75	9.46	Episode-based
01/04/2009	4530	10.04	8.88	Episode-based
01/05/2009	4617	10.24	9.05	Episode-based
01/06/2009	4772	10.52	9.35	Episode-based
01/07/2009	5059	11.19	9.91	Episode-based
01/08/2009	4841	10.72	9.49	Episode-based
01/09/2009	5042	11.16	9.88	Episode-based
01/10/2009	5374	11.88	10.53	Episode-based
01/11/2009	5309	11.72	10.40	Episode-based
01/12/2009	5373	11.89	10.53	Episode-based
01/01/2010	5858	12.82	11.38	Episode-based
01/02/2010	5359	11.72	10.41	Episode-based
01/03/2010	5475	12.00	10.64	Episode-based
01/04/2010	5135	11.25	9.98	Episode-based
01/05/2010	4994	10.92	9.70	Episode-based
01/06/2010	4947	10.78	9.61	Episode-based
01/07/2010	5680	12.41	11.04	Episode-based
01/08/2010	5546	12.10	10.78	Episode-based
01/09/2010	5584	12.19	10.85	Episode-based
01/10/2010	5578	12.19	10.84	Episode-based
01/11/2010	5857	12.80	11.38	Episode-based
01/12/2010	5628	12.29	10.93	Episode-based
01/01/2011	6459	13.82	12.44	Episode-based
01/02/2011	5592	12.02	10.77	Episode-based
01/03/2011	5494	11.82	10.58	Episode-based
01/04/2011	4952	10.68	9.54	Episode-based
01/05/2011	5686	12.21	10.95	Episode-based
01/06/2011	5582	12.06	10.75	Episode-based
01/07/2011	5659	12.16	10.90	Episode-based
01/08/2011	5823	12.55	11.21	Episode-based
01/09/2011	5958	12.84	11.47	Episode-based

01/10/2011	5907	12.71	11.38	Episode-based
01/11/2011	6100	13.12	11.75	Episode-based
01/12/2011	6205	13.39	11.95	Episode-based
01/01/2012	6459	13.69	12.35	Episode-based
01/02/2012	5580	11.85	10.67	Episode-based
01/03/2012	5717	12.11	10.93	Episode-based
01/04/2012	6211	13.18	11.87	Episode-based
01/05/2012	6382	13.48	12.20	Episode-based
01/06/2012	6002	12.70	11.47	Episode-based
01/07/2012	6451	13.70	12.33	Episode-based
01/08/2012	6463	13.66	12.35	Episode-based
01/09/2012	6472	13.69	12.37	Episode-based
01/10/2012	7016	14.88	13.41	Episode-based
01/11/2012	6971	14.79	13.32	Episode-based
01/12/2012	6293	13.34	12.03	Episode-based
01/01/2013	7466	15.61	14.16	Episode-based
01/02/2013	6405	13.36	12.15	Episode-based
01/03/2013	6805	14.21	12.91	Episode-based
01/04/2013	6686	13.94	12.68	Episode-based
01/05/2013	6605	13.78	12.53	Episode-based
01/06/2013	6164	12.86	11.69	Episode-based
01/07/2013	6460	13.45	12.25	Episode-based
01/08/2013	6552	13.64	12.43	Episode-based
01/09/2013	6854	14.29	13.00	Episode-based
01/10/2013	7447	15.53	14.13	Episode-based
01/11/2013	7059	14.73	13.39	Episode-based
01/12/2013	6889	14.34	13.07	Episode-based
01/01/2014	8070	16.54	15.18	Episode-based
01/02/2014	7047	14.46	13.26	Episode-based
01/03/2014	7188	14.72	13.52	Episode-based
01/04/2014	6928	14.20	13.03	Episode-based
01/05/2014	6799	13.92	12.79	Episode-based
01/06/2014	6328	12.96	11.90	Episode-based
01/07/2014	6977	14.32	13.12	Episode-based
01/08/2014	7200	14.72	13.54	Episode-based
01/09/2014	6900	14.11	12.98	Episode-based
01/10/2014	7584	15.55	14.27	Episode-based
01/11/2014	7351	15.07	13.83	Episode-based
01/12/2014	7294	14.92	13.72	Episode-based
01/01/2015	7783	15.76	14.52	Episode-based
01/02/2015	7094	14.33	13.24	Episode-based
01/03/2015	7377	14.92	13.76	Episode-based
01/04/2015	6761	13.66	12.62	Episode-based
01/05/2015	7304	14.76	13.63	Episode-based
01/06/2015	7258	14.69	13.54	Episode-based
01/07/2015	7656	15.47	14.29	Episode-based

01/08/2015	7617	15.38	14.21	Episode-based
01/09/2015	7768	15.68	14.49	Episode-based
01/10/2015	7838	15.86	14.62	Episode-based
01/11/2015	8064	16.32	15.05	Episode-based
01/12/2015	8212	16.65	15.32	Episode-based
01/01/2016	8888	17.73	16.44	Episode-based
01/02/2016	8232	16.42	15.23	Episode-based
01/03/2016	7937	15.80	14.68	Episode-based
01/04/2016	7693	15.36	14.23	Episode-based
01/05/2016	7837	15.58	14.50	Episode-based
01/06/2016	7689	15.33	14.22	Episode-based
01/07/2016	8169	16.27	15.11	Episode-based
01/08/2016	8310	16.56	15.37	Episode-based
01/09/2016	8230	16.39	15.22	Episode-based
01/10/2016	8493	16.91	15.71	Episode-based
01/11/2016	8737	17.39	16.16	Episode-based
01/12/2016	7971	15.89	14.74	Episode-based
01/01/2017	9157	17.96	16.84	Episode-based
01/02/2017	8201	16.10	15.08	Episode-based
01/03/2017	8610	16.89	15.83	Episode-based
01/04/2017	7955	15.61	14.63	Episode-based
01/05/2017	8236	16.14	15.14	Episode-based
01/06/2017	8487	16.66	15.60	Episode-based
01/07/2017	8787	17.23	16.16	Episode-based
01/08/2017	8820	17.25	16.22	Episode-based
01/09/2017	9038	17.72	16.62	Episode-based
01/10/2017	9170	18.01	16.86	Episode-based
01/11/2017	8815	17.28	16.21	Episode-based
01/12/2017	8521	16.71	15.67	Episode-based
01/01/2018	10017	19.37	18.32	Episode-based
01/02/2018	8705	16.83	15.92	Episode-based
01/03/2018	9300	18.01	17.01	Episode-based
01/04/2018	8837	17.11	16.16	Episode-based
01/05/2018	8666	16.79	15.85	Episode-based
01/06/2018	8592	16.60	15.71	Episode-based
01/07/2018	9408	18.21	17.20	Episode-based
01/08/2018	9415	18.20	17.22	Episode-based
01/09/2018	9206	17.80	16.83	Episode-based
01/10/2018	9529	18.48	17.43	Episode-based
01/11/2018	9568	18.53	17.50	Episode-based
01/12/2018	9244	17.88	16.90	Episode-based
01/01/2019	10122	19.24	18.41	Episode-based
01/02/2019	9000	17.13	16.37	Episode-based
01/03/2019	9516	18.12	17.31	Episode-based
01/04/2019	9351	17.81	17.01	Episode-based
01/05/2019	9368	17.81	17.04	Episode-based

01/06/2019	9023	17.20	16.41	Episode-based
01/07/2019	9701	18.45	17.64	Episode-based
01/08/2019	9952	18.93	18.10	Episode-based
01/09/2019	9759	18.54	17.75	Episode-based
01/10/2019	10542	20.05	19.17	Episode-based
01/11/2019	10171	19.36	18.50	Episode-based
01/12/2019	10065	19.15	18.31	Episode-based
01/01/2020	10756	20.29	19.53	Episode-based
01/02/2020	9936	18.76	18.04	Episode-based
01/03/2020	7865	14.87	14.28	Episode-based
01/04/2020	10281	19.43	18.67	Episode-based
01/05/2020	9929	18.77	18.03	Episode-based
01/06/2020	10196	19.22	18.51	Episode-based
01/07/2020	10429	19.70	18.94	Episode-based
01/08/2020	10561	19.90	19.17	Episode-based
01/09/2020	11051	20.85	20.06	Episode-based
01/10/2020	12248	23.11	22.24	Episode-based
01/11/2020	12767	24.12	23.18	Episode-based
01/12/2020	13845	26.15	25.14	Episode-based
01/01/2021	19647	37.01	35.53	Episode-based
01/02/2021	14350	26.98	25.95	Episode-based
01/03/2021	12401	23.33	22.42	Episode-based
01/04/2021	11308	21.27	20.45	Episode-based
01/05/2021	11663	21.88	21.09	Episode-based
01/06/2021	11095	20.91	20.06	Episode-based
01/07/2021	12621	23.71	22.82	Episode-based
01/08/2021	12661	23.78	22.89	Episode-based
01/09/2021	12848	24.17	23.23	Episode-based
01/10/2021	13098	24.65	23.68	Episode-based
01/11/2021	13174	24.80	23.82	Episode-based
01/12/2021	13336	25.07	24.12	Episode-based
01/01/2022	13229	24.64	23.69	Episode-based
01/02/2022	11463	21.33	20.53	Episode-based
01/03/2022	11585	21.57	20.74	Episode-based
01/04/2022	10924	20.34	19.56	Episode-based
01/05/2022	11262	20.99	20.17	Episode-based
01/06/2022	11142	20.73	19.95	Episode-based
01/07/2022	11272	21.00	20.18	Episode-based
01/08/2022	12328	22.95	22.07	Episode-based
01/09/2022	12359	23.03	22.13	Episode-based
01/10/2022	12576	23.45	22.52	Episode-based
01/11/2022	12360	23.05	22.13	Episode-based
01/12/2022	11575	21.54	20.73	Episode-based
01/01/2023	12831	23.68	22.75	Episode-based
01/02/2023	10185	18.80	18.05	Episode-based
01/03/2023	11382	21.02	20.18	Episode-based

01/04/2023	10806	19.96	19.16	Episode-based
01/05/2023	11090	20.49	19.66	Episode-based
01/06/2023	10685	19.74	18.94	Episode-based
01/07/2023	11444	21.11	20.29	Episode-based
01/08/2023	11754	21.70	20.84	Episode-based
01/09/2023	11719	21.67	20.77	Episode-based
01/10/2023	13039	24.04	23.11	Episode-based
01/11/2023	12895	23.84	22.86	Episode-based
01/12/2023	12495	23.08	22.15	Episode-based
01/01/2024	12948	23.92	22.95	Episode-based
01/02/2024	11929	21.99	21.15	Episode-based
01/03/2024	11422	21.08	20.25	Episode-based
01/04/2024	11657	21.52	20.66	Episode-based
01/05/2024	11782	21.74	20.89	Episode-based
01/06/2024	10927	20.18	19.37	Episode-based
01/07/2024	12156	22.42	21.55	Episode-based
01/08/2024	11869	21.94	21.04	Episode-based
01/09/2024	11643	21.50	20.64	Episode-based
01/10/2024	12543	23.12	22.23	Episode-based
01/11/2024	11705	21.63	20.75	Episode-based
01/12/2024	10783	19.91	19.11	Episode-based
01/04/2008	2398	5.37	4.73	Person-based
01/05/2008	2275	5.08	4.49	Person-based
01/06/2008	2347	5.26	4.63	Person-based
01/07/2008	2322	5.20	4.58	Person-based
01/08/2008	2376	5.30	4.69	Person-based
01/09/2008	2458	5.50	4.85	Person-based
01/10/2008	2593	5.82	5.12	Person-based
01/11/2008	2441	5.48	4.82	Person-based
01/12/2008	2494	5.61	4.92	Person-based
01/01/2009	2874	6.36	5.63	Person-based
01/02/2009	2421	5.37	4.74	Person-based
01/03/2009	2532	5.62	4.96	Person-based
01/04/2009	2412	5.33	4.73	Person-based
01/05/2009	2380	5.28	4.66	Person-based
01/06/2009	2451	5.40	4.80	Person-based
01/07/2009	2622	5.80	5.14	Person-based
01/08/2009	2517	5.57	4.93	Person-based
01/09/2009	2618	5.79	5.13	Person-based
01/10/2009	2775	6.12	5.44	Person-based
01/11/2009	2712	5.98	5.31	Person-based
01/12/2009	2792	6.18	5.47	Person-based
01/01/2010	3005	6.57	5.84	Person-based
01/02/2010	2726	5.96	5.30	Person-based
01/03/2010	2757	6.05	5.36	Person-based
01/04/2010	2531	5.53	4.92	Person-based

01/05/2010	2480	5.41	4.82	Person-based
01/06/2010	2504	5.46	4.87	Person-based
01/07/2010	2805	6.11	5.45	Person-based
01/08/2010	2726	5.95	5.30	Person-based
01/09/2010	2789	6.08	5.42	Person-based
01/10/2010	2767	6.05	5.38	Person-based
01/11/2010	2933	6.42	5.70	Person-based
01/12/2010	2804	6.12	5.45	Person-based
01/01/2011	3198	6.86	6.16	Person-based
01/02/2011	2778	5.98	5.35	Person-based
01/03/2011	2715	5.84	5.23	Person-based
01/04/2011	2387	5.14	4.60	Person-based
01/05/2011	2761	5.95	5.32	Person-based
01/06/2011	2718	5.86	5.23	Person-based
01/07/2011	2788	6.00	5.37	Person-based
01/08/2011	2831	6.11	5.45	Person-based
01/09/2011	2918	6.27	5.62	Person-based
01/10/2011	2834	6.09	5.46	Person-based
01/11/2011	2938	6.33	5.66	Person-based
01/12/2011	3060	6.61	5.89	Person-based
01/01/2012	3223	6.83	6.16	Person-based
01/02/2012	2781	5.89	5.32	Person-based
01/03/2012	2741	5.81	5.24	Person-based
01/04/2012	2917	6.20	5.58	Person-based
01/05/2012	3033	6.42	5.80	Person-based
01/06/2012	2869	6.06	5.48	Person-based
01/07/2012	3085	6.54	5.90	Person-based
01/08/2012	3048	6.43	5.83	Person-based
01/09/2012	3052	6.45	5.83	Person-based
01/10/2012	3334	7.06	6.37	Person-based
01/11/2012	3212	6.80	6.14	Person-based
01/12/2012	3001	6.37	5.74	Person-based
01/01/2013	3612	7.54	6.85	Person-based
01/02/2013	3077	6.41	5.84	Person-based
01/03/2013	3232	6.73	6.13	Person-based
01/04/2013	3122	6.49	5.92	Person-based
01/05/2013	3127	6.52	5.93	Person-based
01/06/2013	2884	6.01	5.47	Person-based
01/07/2013	3021	6.29	5.73	Person-based
01/08/2013	3087	6.42	5.86	Person-based
01/09/2013	3105	6.47	5.89	Person-based
01/10/2013	3491	7.27	6.62	Person-based
01/11/2013	3319	6.93	6.30	Person-based
01/12/2013	3265	6.80	6.19	Person-based
01/01/2014	3787	7.76	7.12	Person-based
01/02/2014	3376	6.92	6.35	Person-based

01/03/2014	3261	6.67	6.13	Person-based
01/04/2014	3157	6.47	5.94	Person-based
01/05/2014	3097	6.34	5.83	Person-based
01/06/2014	2882	5.90	5.42	Person-based
01/07/2014	3207	6.57	6.03	Person-based
01/08/2014	3350	6.84	6.30	Person-based
01/09/2014	3255	6.65	6.12	Person-based
01/10/2014	3529	7.24	6.64	Person-based
01/11/2014	3414	7.00	6.42	Person-based
01/12/2014	3365	6.88	6.33	Person-based
01/01/2015	3718	7.53	6.94	Person-based
01/02/2015	3211	6.49	5.99	Person-based
01/03/2015	3449	6.99	6.44	Person-based
01/04/2015	3076	6.23	5.74	Person-based
01/05/2015	3327	6.73	6.21	Person-based
01/06/2015	3170	6.41	5.91	Person-based
01/07/2015	3334	6.73	6.22	Person-based
01/08/2015	3339	6.74	6.23	Person-based
01/09/2015	3518	7.10	6.56	Person-based
01/10/2015	3426	6.92	6.39	Person-based
01/11/2015	3506	7.09	6.54	Person-based
01/12/2015	3683	7.45	6.87	Person-based
01/01/2016	3991	7.95	7.38	Person-based
01/02/2016	3668	7.31	6.78	Person-based
01/03/2016	3615	7.19	6.69	Person-based
01/04/2016	3351	6.68	6.20	Person-based
01/05/2016	3430	6.82	6.34	Person-based
01/06/2016	3284	6.55	6.07	Person-based
01/07/2016	3502	6.97	6.48	Person-based
01/08/2016	3584	7.14	6.63	Person-based
01/09/2016	3664	7.29	6.78	Person-based
01/10/2016	3729	7.40	6.90	Person-based
01/11/2016	3779	7.52	6.99	Person-based
01/12/2016	3500	6.98	6.47	Person-based
01/01/2017	4088	8.02	7.52	Person-based
01/02/2017	3614	7.09	6.64	Person-based
01/03/2017	3831	7.52	7.04	Person-based
01/04/2017	3343	6.55	6.15	Person-based
01/05/2017	3608	7.07	6.63	Person-based
01/06/2017	3575	7.02	6.57	Person-based
01/07/2017	3763	7.37	6.92	Person-based
01/08/2017	3846	7.52	7.07	Person-based
01/09/2017	3923	7.69	7.21	Person-based
01/10/2017	3927	7.70	7.22	Person-based
01/11/2017	3721	7.29	6.84	Person-based
01/12/2017	3702	7.26	6.81	Person-based

01/01/2018	4449	8.59	8.14	Person-based
01/02/2018	3811	7.37	6.97	Person-based
01/03/2018	4119	7.97	7.53	Person-based
01/04/2018	3791	7.33	6.93	Person-based
01/05/2018	3561	6.89	6.51	Person-based
01/06/2018	3604	6.96	6.59	Person-based
01/07/2018	3807	7.37	6.96	Person-based
01/08/2018	3956	7.64	7.23	Person-based
01/09/2018	3902	7.53	7.14	Person-based
01/10/2018	4010	7.76	7.33	Person-based
01/11/2018	4026	7.78	7.36	Person-based
01/12/2018	3908	7.55	7.15	Person-based
01/01/2019	4338	8.24	7.89	Person-based
01/02/2019	3845	7.32	6.99	Person-based
01/03/2019	3967	7.54	7.21	Person-based
01/04/2019	3766	7.17	6.85	Person-based
01/05/2019	3825	7.27	6.96	Person-based
01/06/2019	3641	6.93	6.62	Person-based
01/07/2019	3971	7.55	7.22	Person-based
01/08/2019	4024	7.65	7.32	Person-based
01/09/2019	4036	7.68	7.34	Person-based
01/10/2019	4361	8.30	7.93	Person-based
01/11/2019	4262	8.11	7.75	Person-based
01/12/2019	4228	8.05	7.69	Person-based
01/01/2020	4514	8.51	8.20	Person-based
01/02/2020	4028	7.60	7.31	Person-based
01/03/2020	3305	6.24	6.00	Person-based
01/04/2020	4487	8.48	8.15	Person-based
01/05/2020	4215	7.96	7.65	Person-based
01/06/2020	4242	7.99	7.70	Person-based
01/07/2020	4328	8.17	7.86	Person-based
01/08/2020	4348	8.20	7.89	Person-based
01/09/2020	4472	8.43	8.12	Person-based
01/10/2020	5179	9.77	9.40	Person-based
01/11/2020	5446	10.29	9.89	Person-based
01/12/2020	5957	11.26	10.82	Person-based
01/01/2021	8897	16.77	16.09	Person-based
01/02/2021	6235	11.73	11.27	Person-based
01/03/2021	5359	10.08	9.69	Person-based
01/04/2021	4686	8.81	8.47	Person-based
01/05/2021	4709	8.84	8.52	Person-based
01/06/2021	4458	8.39	8.06	Person-based
01/07/2021	5164	9.69	9.34	Person-based
01/08/2021	5364	10.06	9.70	Person-based
01/09/2021	5410	10.17	9.78	Person-based
01/10/2021	5438	10.23	9.83	Person-based

01/11/2021	5579	10.51	10.09	Person-based
01/12/2021	5751	10.81	10.40	Person-based
01/01/2022	5625	10.48	10.07	Person-based
01/02/2022	4870	9.07	8.72	Person-based
01/03/2022	4851	9.04	8.69	Person-based
01/04/2022	4503	8.37	8.06	Person-based
01/05/2022	4658	8.69	8.34	Person-based
01/06/2022	4512	8.40	8.08	Person-based
01/07/2022	4611	8.60	8.26	Person-based
01/08/2022	4997	9.31	8.95	Person-based
01/09/2022	5004	9.32	8.96	Person-based
01/10/2022	5109	9.53	9.15	Person-based
01/11/2022	5092	9.51	9.12	Person-based
01/12/2022	4804	8.94	8.60	Person-based
01/01/2023	5443	10.04	9.65	Person-based
01/02/2023	4161	7.67	7.38	Person-based
01/03/2023	4642	8.57	8.23	Person-based
01/04/2023	4273	7.89	7.57	Person-based
01/05/2023	4349	8.03	7.71	Person-based
01/06/2023	4223	7.80	7.49	Person-based
01/07/2023	4502	8.30	7.98	Person-based
01/08/2023	4625	8.53	8.20	Person-based
01/09/2023	4704	8.69	8.34	Person-based
01/10/2023	5211	9.60	9.24	Person-based
01/11/2023	5144	9.50	9.12	Person-based
01/12/2023	5012	9.26	8.88	Person-based
01/01/2024	5272	9.73	9.35	Person-based
01/02/2024	4829	8.91	8.56	Person-based
01/03/2024	4703	8.68	8.34	Person-based
01/04/2024	4582	8.46	8.12	Person-based
01/05/2024	4555	8.40	8.07	Person-based
01/06/2024	4247	7.85	7.53	Person-based
01/07/2024	4645	8.57	8.23	Person-based
01/08/2024	4594	8.48	8.14	Person-based
01/09/2024	4455	8.21	7.90	Person-based
01/10/2024	4913	9.05	8.71	Person-based
01/11/2024	4583	8.47	8.12	Person-based
01/12/2024	4351	8.04	7.71	Person-based

eTable 3 Prior Pulmonary Embolism History for Patients Admitted in 2019, Demonstrating the Adequacy of a Five-Year Look-back Window (n = 56 143)

	**Count**	**% of 2019 cases**
**Prior PE**	**13 974**	**24.9 %**
Interval since last PE record		
≤ 3 months	6 770	12.1 %
3–6 months	1 215	2.2 %
6–12 months	1 397	2.5 %
1–3 years	2 009	3.6 %
3–5 years	883	1.6 %
5–10 years	1 145	2.0 %
10–17 years	555	1.0 %
**No prior PE**	**42 169**	**75.1 %**

eFigure 1 Flow diagram of individuals included in the analysis with first-time hospital-recorded PE from April 2008 through December 2024.

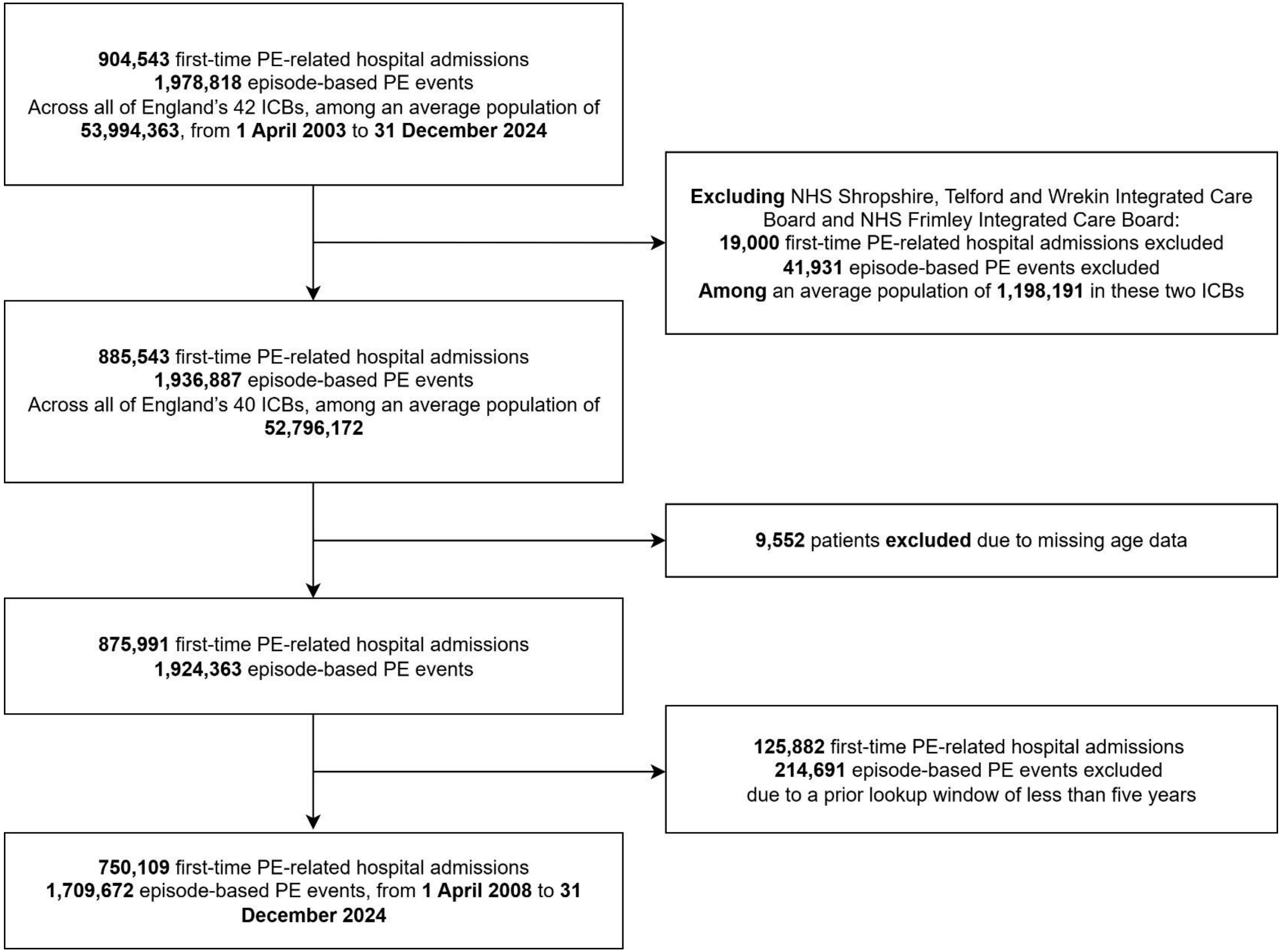

eFigure 2 First-time hospital-recorded PE rates by age group (age-standardised), April 2008 to December 2024

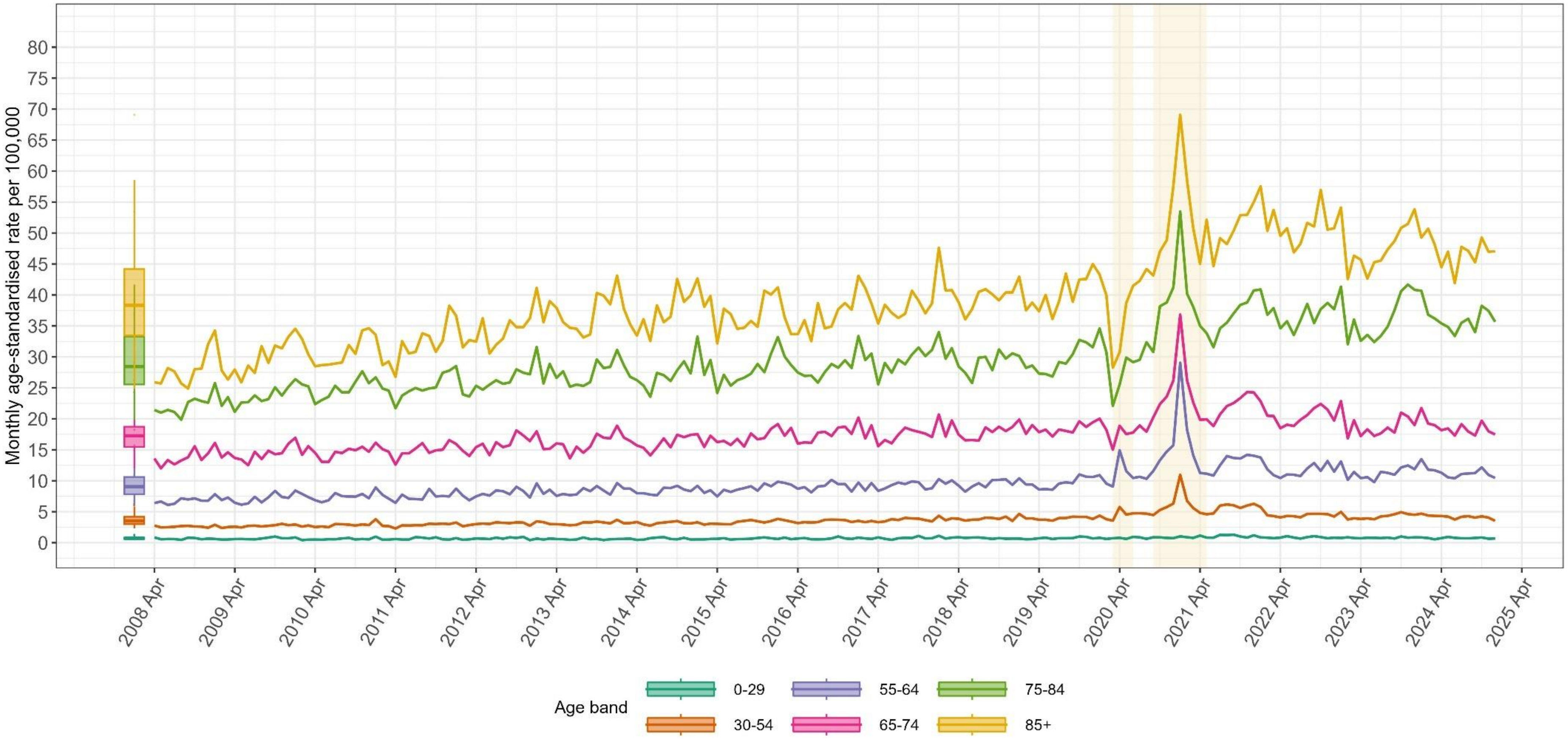

The numerator is the number of first-time admissions to hospital with PE per patient, in any diagnostic position, grouped by broader age group); age-standardised using the 2013 European Standard Population. The denominator is the total baseline population based on mid-year population estimates from the Office for National Statistics. Boxplots show the historical average age-standardised PE rate from April 2008 to December 2024 (median and interquartile range). The shaded area indicates the first and second waves of the coronavirus pandemic in England.

eFigure 3 **First-time hospital-recorded PE rates by sex (age-standardised), April 2008 to December 2024**

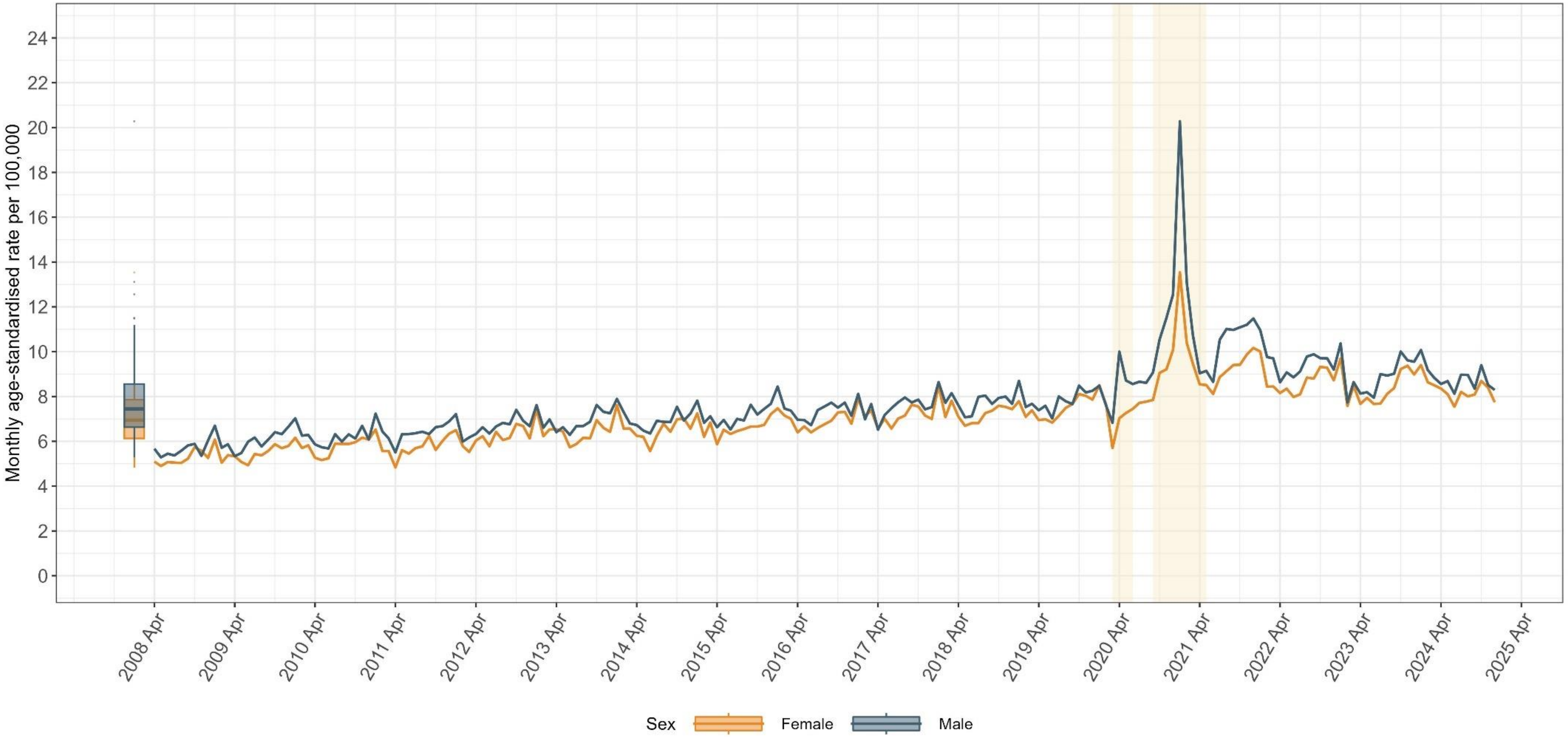

The numerator is the number of first-time admissions to hospital with PE per patient, in any diagnostic position, grouped by sex; age-standardised using the 2013 European Standard Population. The denominator is the total baseline population based on mid-year population estimates from the Office for National Statistics. Boxplots show the historical average age-standardised PE rate from April 2008 to December 2024 (median and interquartile range). The shaded area indicates the first and second waves of the coronavirus pandemic in England.

eFigure 4 First-time hospital-recorded PE rates by the 9 regions (age-standardised), April 2008 to December 2024

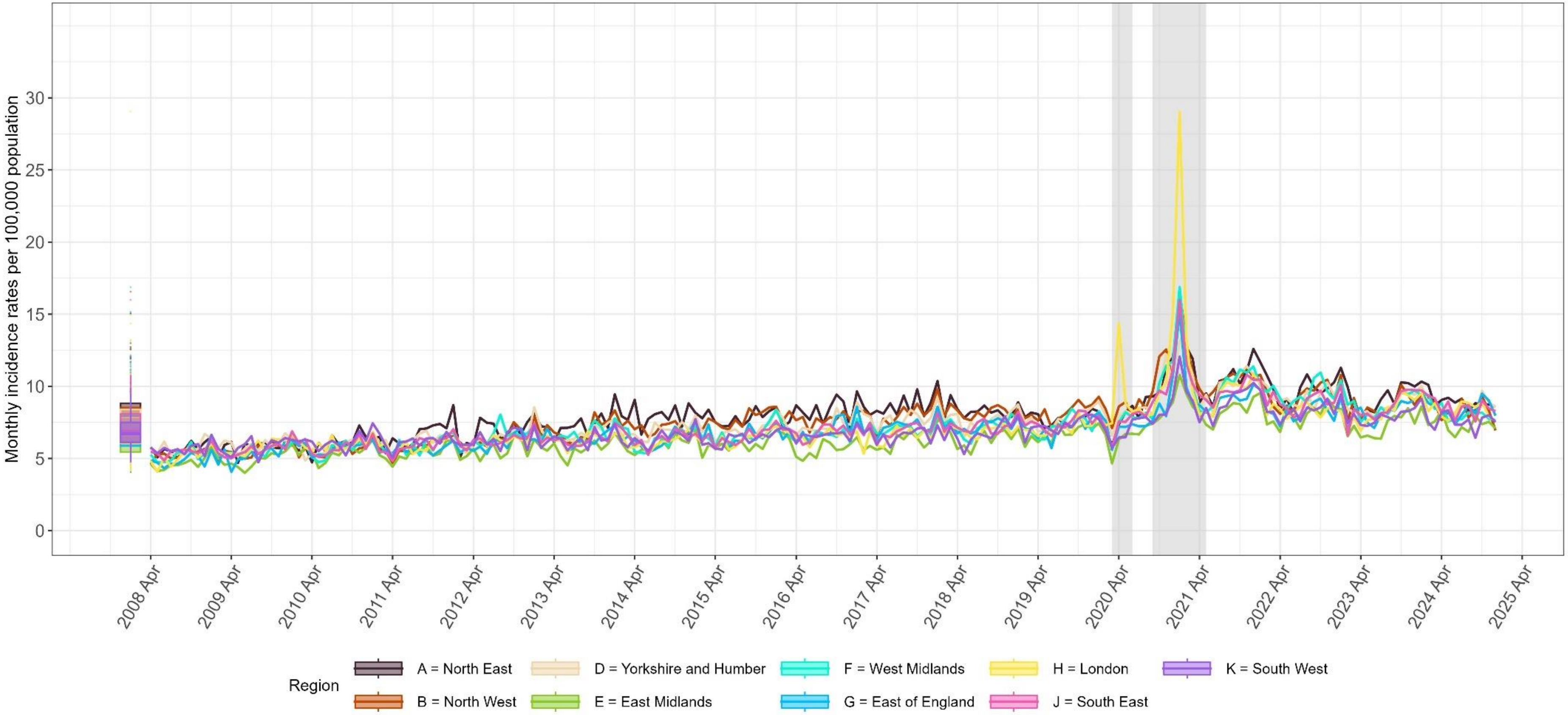

The numerator is the number of first-time admissions to hospital with PE per patient, in any diagnostic position, grouped by region; age-standardised using the 2013 European Standard Population. The denominator is the total baseline population based on mid-year population estimates from the Office for National Statistics. Boxplots show the historical average age-standardised PE rate from April 2008 to December 2024 (median and interquartile range). The shaded area indicates the first and second waves of the coronavirus pandemic in England.

eFigure 5 First-time hospital-recorded PE rates by IMD quintile (age-standardised), April 2008 to December 2024

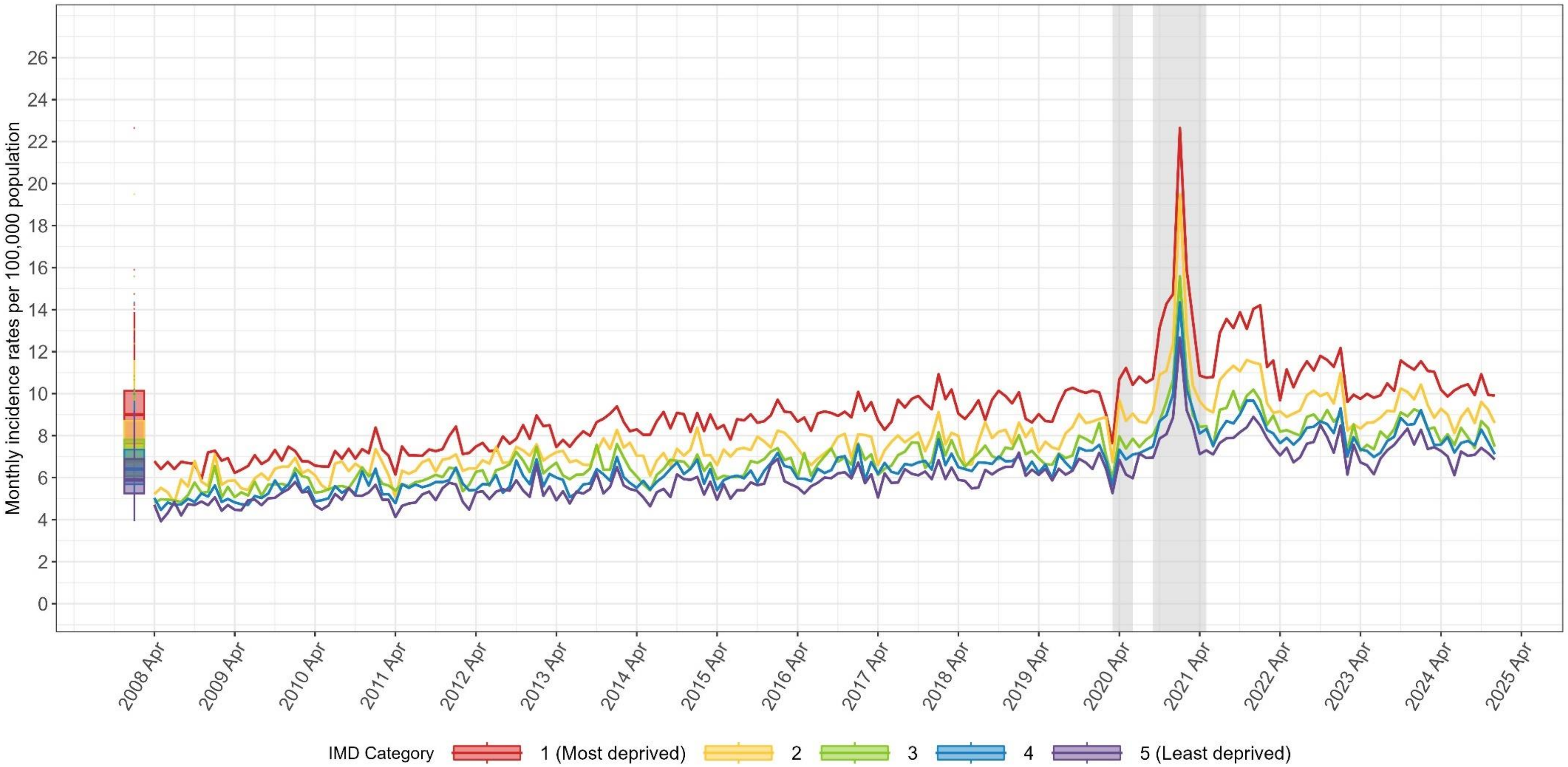

The numerator is the number of first-time admissions to hospital with PE per patient, in any diagnostic position, grouped by IMD (Index of Multiple Deprivation, IMD quintile measured from patient-level address); age-standardised using the 2013 European Standard Population. The denominator is the total baseline population based on mid-year population estimates from the Office for National Statistics. Boxplots show the historical average age-standardised PE rate from April 2008 to December 2024 (median and interquartile range). The shaded area indicates the first and second waves of the coronavirus pandemic in England.

eFigure 6 **First-time, episode-based hospital-recorded PE rates (age-standardised), April 2008 to December 2024**

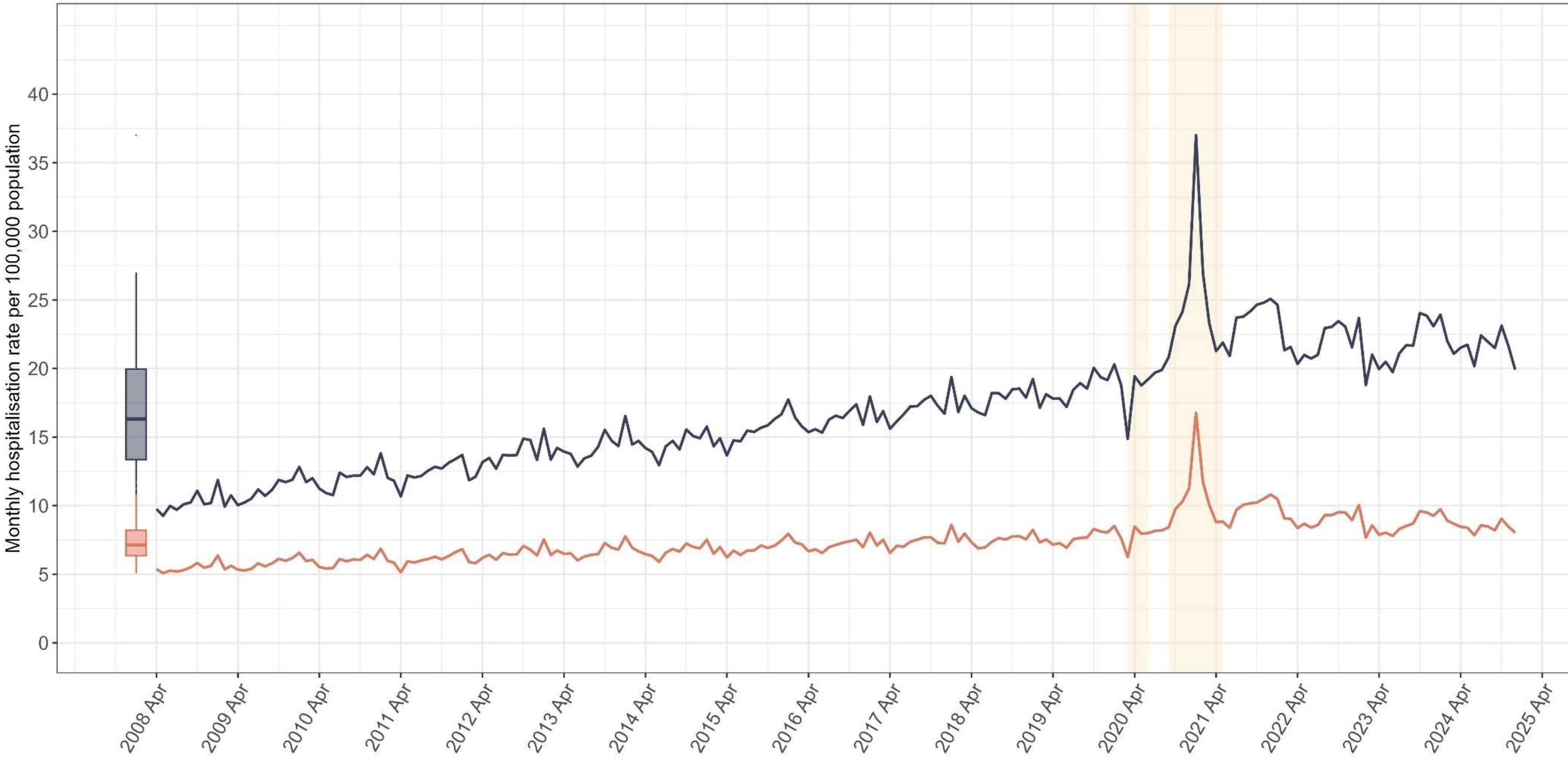

The numerator is the number of first-time admissions to hospital with PE per patient, in any diagnostic position, age-standardised using the 2013 European Standard Population. The denominator is the total baseline population based on mid-year population estimates from the Office for National Statistics. Boxplots show the historical average age-standardised PE rate from April 2008 to December 2024 (median and interquartile range). The shaded area indicates the first and second waves of the coronavirus pandemic in England.

eFigure 7 **First-time, episode-based hospital-recorded PE rates (crude), April 2008 to December 2024**

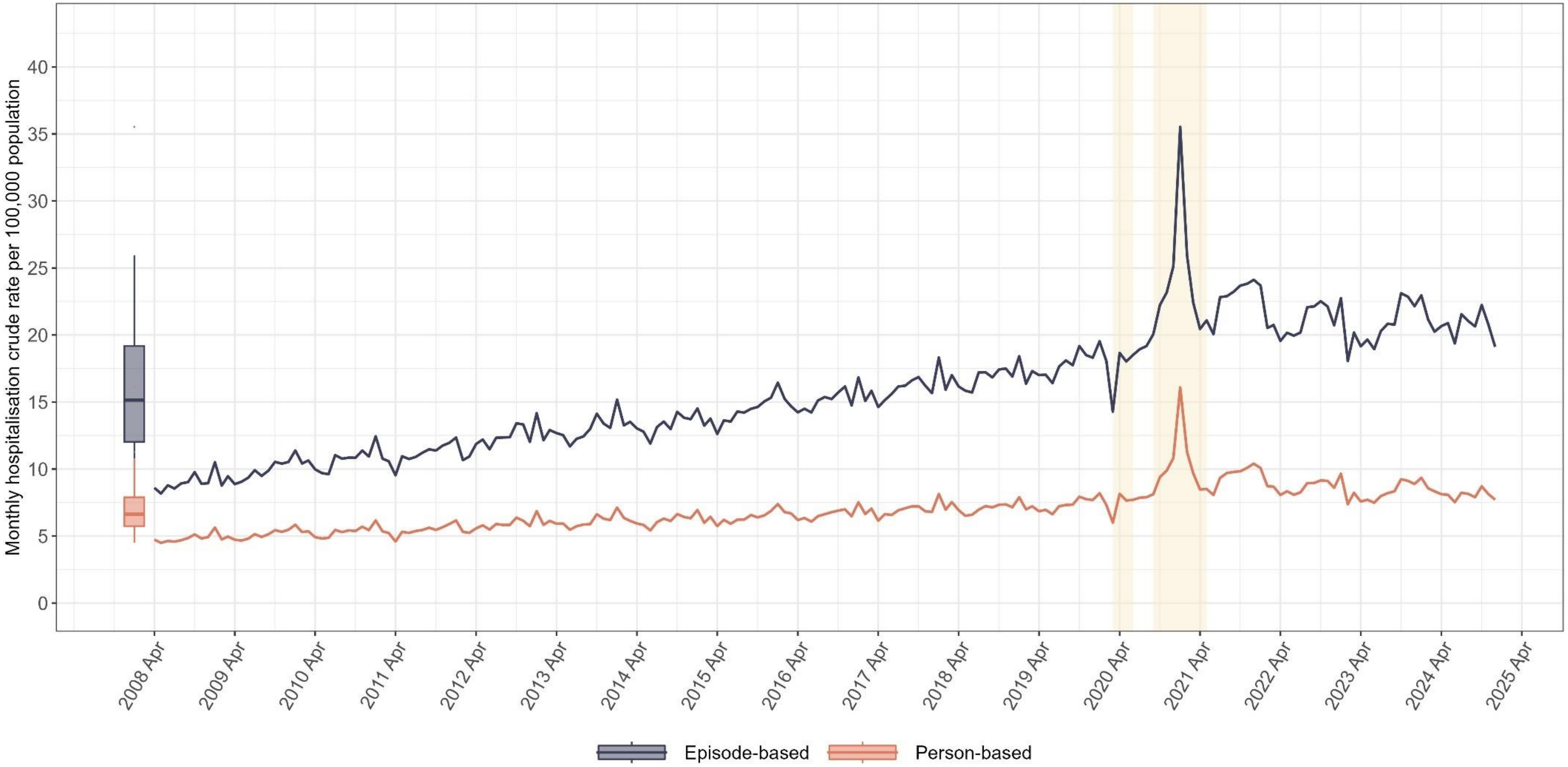

The numerator is the number of first-time admissions to hospital with PE per patient, in any diagnostic position. The denominator is the total baseline population based on mid-year population estimates from the Office for National Statistics. Boxplots show the historical average crude PE rate from April 2008 to December 2024 (median and interquartile range). The shaded area indicates the first and second waves of the coronavirus pandemic in England.

eFigure 8 Effect of the COVID-19 pandemic on first-time PE incidence rates, with and without co-existing COVID-19, comparing post-pandemic months (March 2020 to December 2024) with pre-pandemic trend (April 2008 to February 2020); cohort with a consistent 5-year look-back history

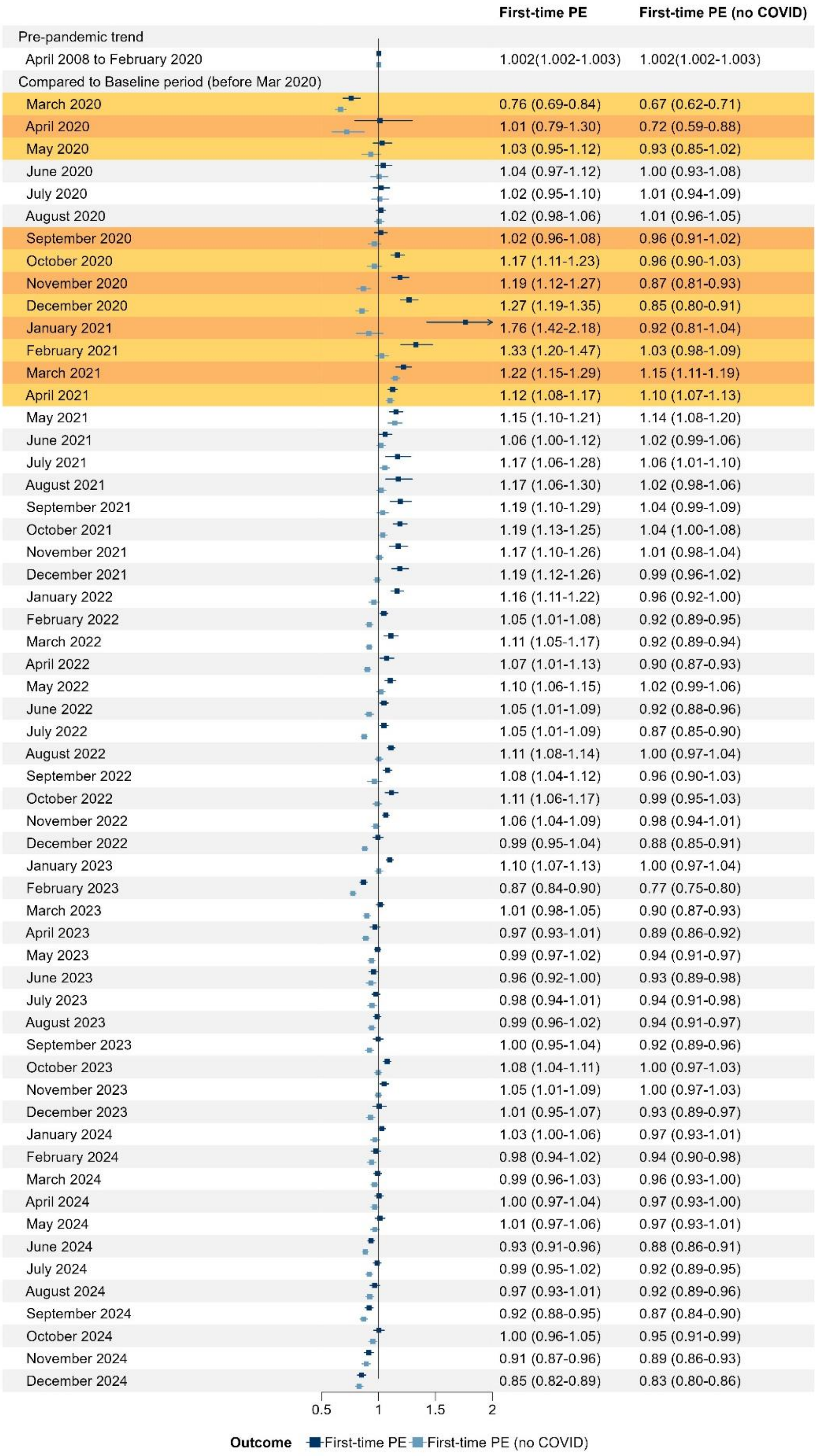

The model is adjusted for age (5-year age groups) and sex, with month treated as a continuous variable and each month from March 2020 onwards treated as a categorical variable. The IRR in "pre-pandemic trends" represents the long-term trend before February 2020. The orange shaded area indicates the first and second waves of the coronavirus pandemic in England.

eFigure 9 First-time hospital-recorded PE rates: comparison of cohorts with (i) a minimum 5-year look-back history (main study population) and (ii) a consistent 5-year look-back history, April 2008–December 2024

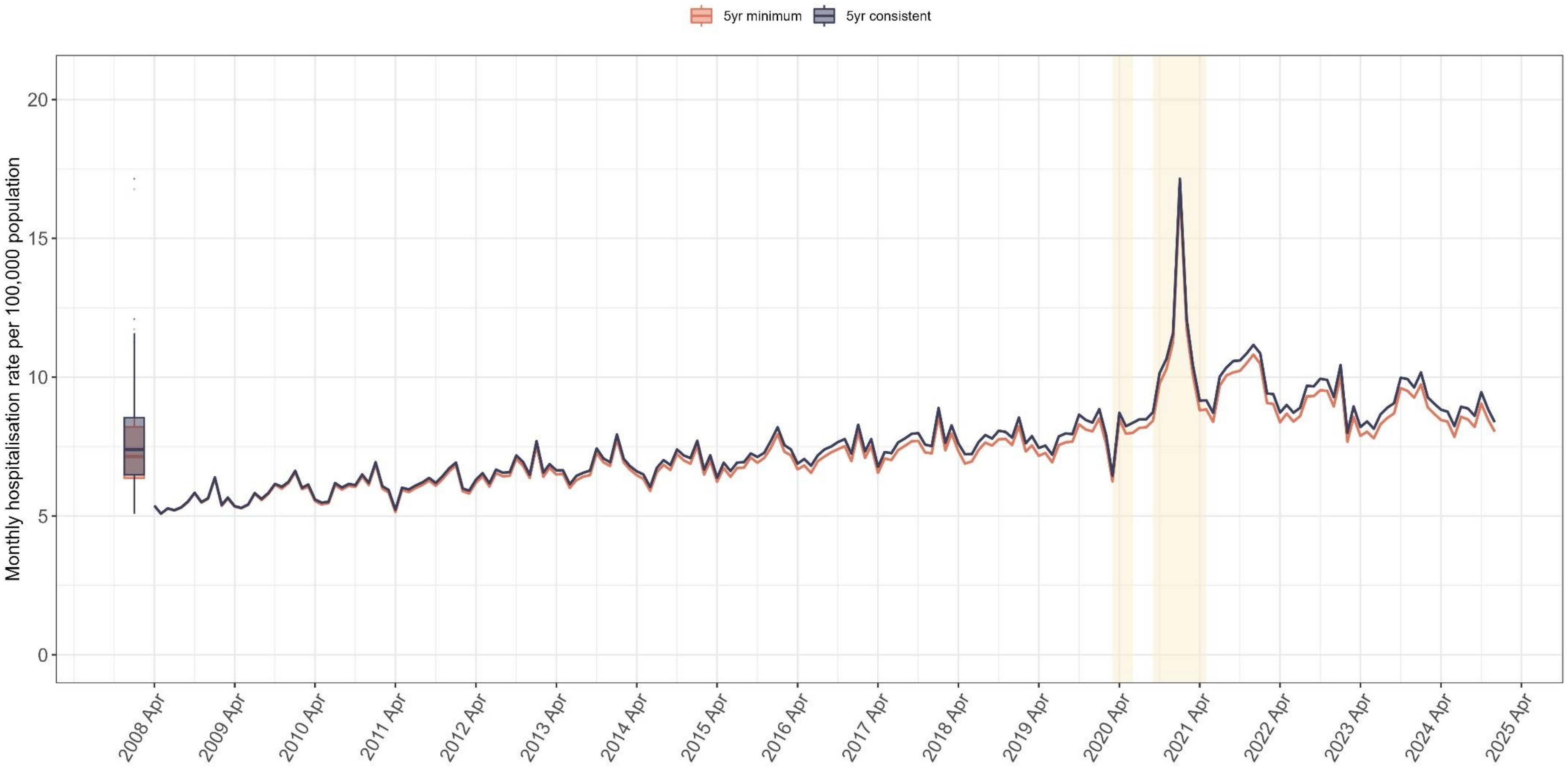

The numerator is the number of first-time admissions to hospital with PE per patient, in any diagnostic position. The denominator is the total baseline population based on mid-year population estimates from the Office for National Statistics. Boxplots show the historical average crude PE rate from April 2008 to December 2024 (median and interquartile range). The shaded area indicates the first and second waves of the coronavirus pandemic in England.

eFigure 10 Effect of the COVID-19 pandemic on first-time PE incidence rates, with and without co-existing COVID-19, comparing post-pandemic months (March 2020 to December 2024) with pre-pandemic trend (April 2008 to February 2020); COVID diagnosis using ICD-10 U07.1 and U07.2

	First-time PE	First-time PE (no COVID)
Pre-pandemic trend		
April 2008 to February 2020	1.002(1.002-1.002)	1.002(1.002-1.002)
Compared to Baseline period (before Mar 2020)		
March 2020	0.77 (0.69-0.85)	0.66 (0.61-0.71)
April 2020	1.03 (0.79-1.33)	0.64 (0.53-0.78)
May 2020	1.04 (0.97-1.13)	0.89 (0.81-0.97)
June 2020	1.04 (0.97-1.12)	0.98 (0.91-1.06)
July 2020	1.03 (0.96-1.11)	1.01 (0.94-1.09)
August 2020	1.03 (0.99-1.08)	1.01 (0.97-1.06)
September 2020	1.03 (0.98-1.09)	0.96 (0.91-1.02)
October 2020	1.17 (1.11-1.24)	0.95 (0.89-1.01)
November 2020	1.20 (1.12-1.28)	0.85 (0.79-0.91)
December 2020	1.29 (1.21-1.37)	0.84 (0.79-0.90)
January 2021	1.81 (1.46-2.24)	0.90 (0.80-1.01)
February 2021	1.35 (1.21-1.50)	1.02 (0.97-1.07)
March 2021	1.23 (1.17-1.31)	1.15 (1.11-1.19)
April 2021	1.13 (1.09-1.18)	1.10 (1.07-1.14)
May 2021	1.17 (1.11-1.23)	1.15 (1.09-1.22)
June 2021	1.07 (1.01-1.13)	1.02 (0.99-1.06)
July 2021	1.18 (1.07-1.31)	1.06 (1.02-1.11)
August 2021	1.19 (1.08-1.32)	1.03 (0.99-1.07)
September 2021	1.20 (1.10-1.30)	1.03 (0.98-1.09)
October 2021	1.20 (1.13-1.27)	1.04 (1.00-1.08)
November 2021	1.19 (1.11-1.28)	1.01 (0.98-1.05)
December 2021	1.21 (1.14-1.29)	0.99 (0.96-1.03)
January 2022	1.18 (1.13-1.24)	0.97 (0.93-1.01)
February 2022	1.06 (1.03-1.10)	0.93 (0.90-0.96)
March 2022	1.12 (1.06-1.19)	0.93 (0.90-0.95)
April 2022	1.08 (1.02-1.14)	0.91 (0.88-0.94)
May 2022	1.12 (1.07-1.17)	1.04 (1.00-1.08)
June 2022	1.06 (1.03-1.10)	0.93 (0.89-0.97)
July 2022	1.07 (1.03-1.11)	0.89 (0.86-0.91)
August 2022	1.12 (1.09-1.16)	1.01 (0.98-1.05)
September 2022	1.09 (1.06-1.13)	0.97 (0.90-1.04)
October 2022	1.12 (1.07-1.18)	0.99 (0.96-1.03)
November 2022	1.08 (1.05-1.11)	0.98 (0.95-1.02)
December 2022	1.01 (0.97-1.06)	0.89 (0.86-0.91)
January 2023	1.12 (1.08-1.15)	1.01 (0.98-1.05)
February 2023	0.88 (0.85-0.91)	0.78 (0.75-0.81)
March 2023	1.02 (0.99-1.06)	0.90 (0.87-0.94)
April 2023	0.98 (0.94-1.02)	0.90 (0.87-0.93)
May 2023	1.00 (0.98-1.03)	0.95 (0.91-0.98)
June 2023	0.97 (0.93-1.01)	0.94 (0.90-0.99)
July 2023	0.99 (0.96-1.03)	0.95 (0.92-0.99)
August 2023	1.00 (0.97-1.03)	0.95 (0.92-0.98)
September 2023	1.01 (0.97-1.05)	0.93 (0.89-0.97)
October 2023	1.09 (1.06-1.13)	1.01 (0.98-1.04)
November 2023	1.06 (1.02-1.10)	1.01 (0.98-1.04)
December 2023	1.02 (0.97-1.09)	0.95 (0.91-0.98)
January 2024	1.04 (1.01-1.08)	0.98 (0.94-1.02)
February 2024	0.99 (0.95-1.04)	0.96 (0.92-1.00)
March 2024	1.01 (0.97-1.05)	0.98 (0.95-1.02)
April 2024	1.02 (0.98-1.05)	0.98 (0.94-1.01)
May 2024	1.03 (0.99-1.08)	0.99 (0.95-1.03)
June 2024	0.94 (0.92-0.97)	0.89 (0.87-0.92)
July 2024	1.01 (0.97-1.04)	0.93 (0.91-0.96)
August 2024	0.98 (0.94-1.03)	0.93 (0.90-0.97)
September 2024	0.93 (0.89-0.96)	0.88 (0.84-0.91)
October 2024	1.01 (0.97-1.05)	0.96 (0.93-0.99)
November 2024	0.93 (0.89-0.97)	0.91 (0.87-0.94)
December 2024	0.87 (0.83-0.91)	0.85 (0.81-0.88)

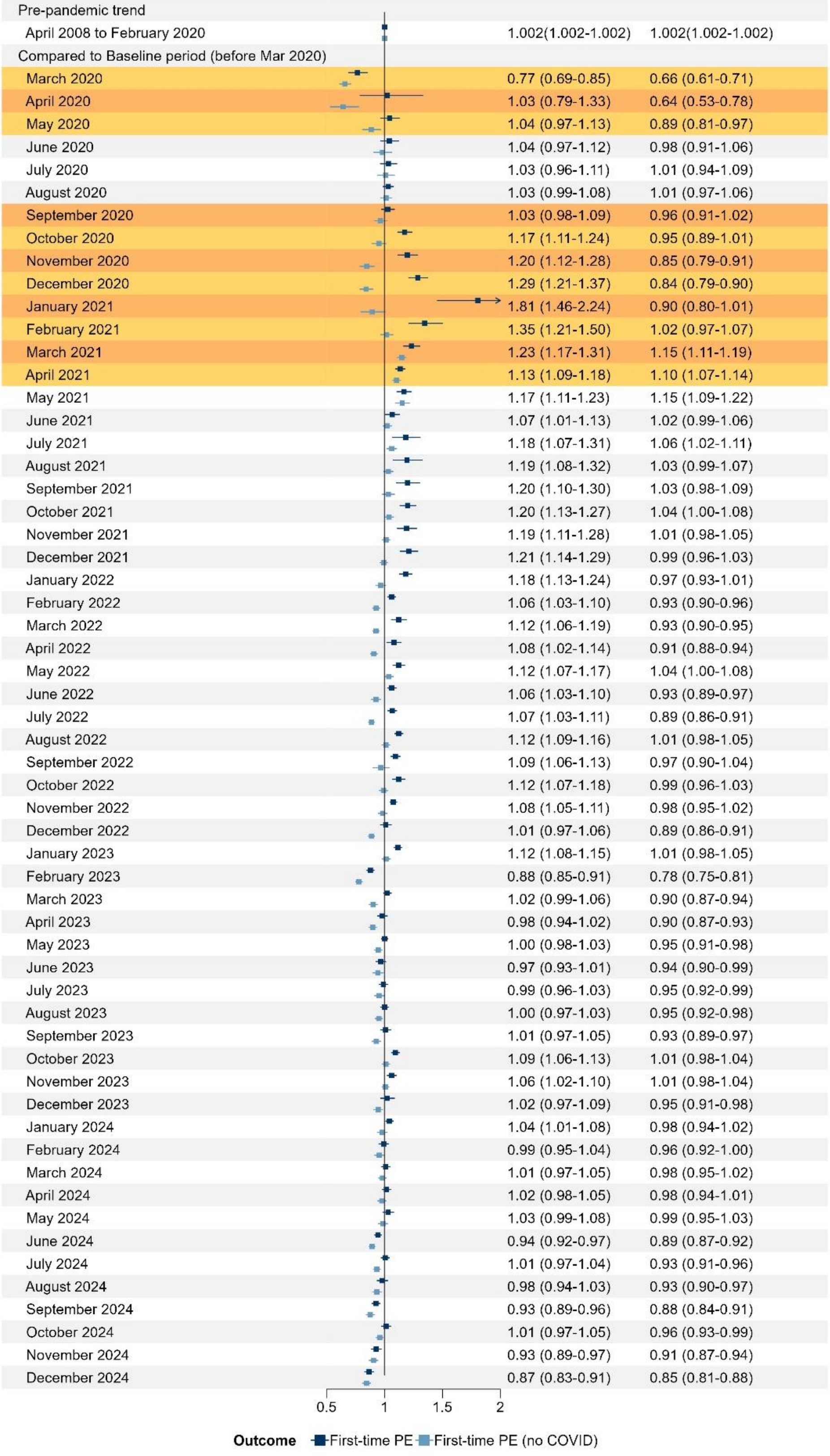

eFigure 11 First-time hospital-recorded PE rates by diagnosis position, any position (blue) vs primary (first) position (green), April 2008 – December 2024

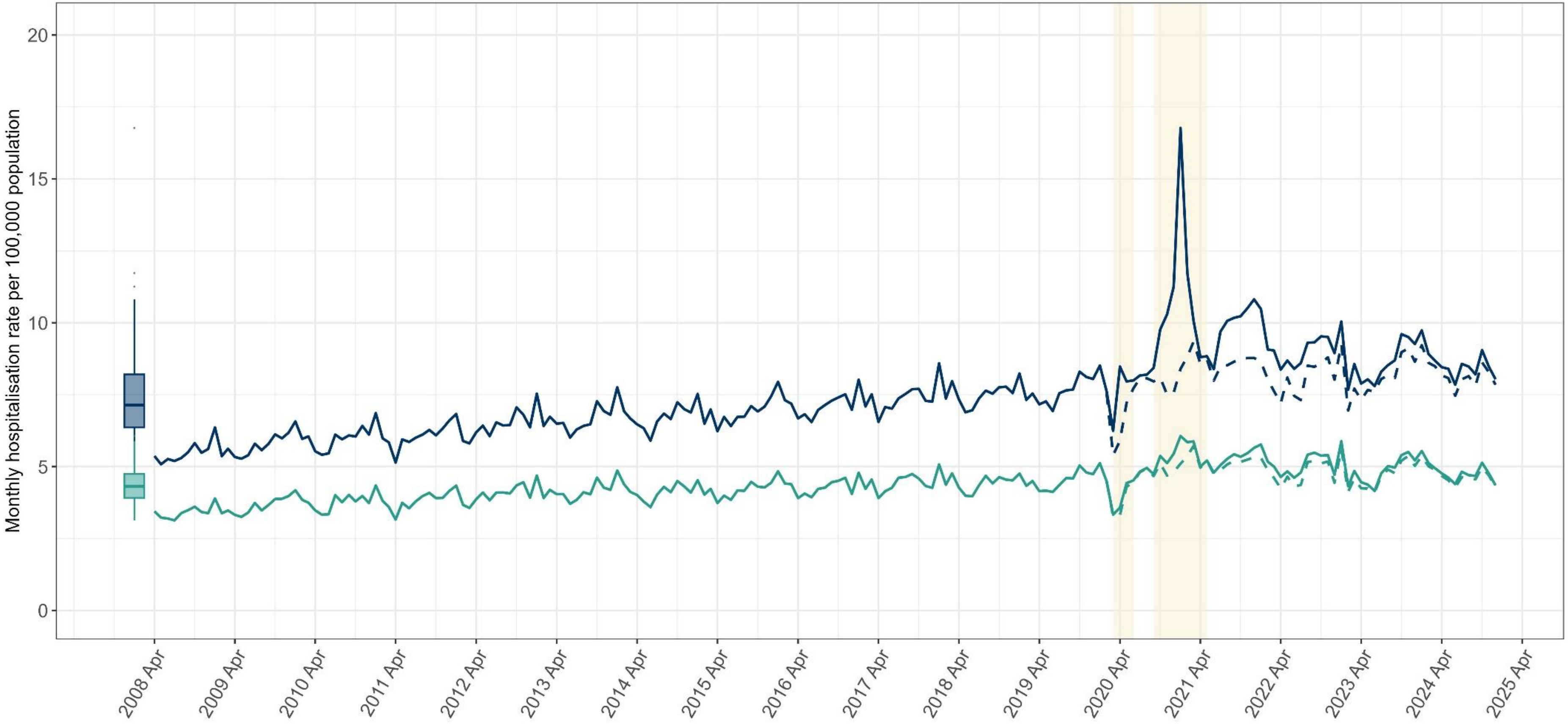

The numerator is the number of first-time admissions to hospital with PE per patient, in any diagnostic position vs in primary position, age-standardised using the 2013 European Standard Population. The denominator is the total baseline population based on mid-year population estimates from the Office for National Statistics. Boxplots show the historical average age-standardised PE rate from April 2008 to December 2024 (median and interquartile range). The shaded area indicates the first and second waves of the coronavirus pandemic in England.

eFigure 12 Effect of the COVID-19 pandemic on first-time PE incidence rates, with and without co-existing COVID-19, comparing post-pandemic months (March 2020 to December 2024) with pre-pandemic trend (April 2008 to February 2020); PE in primary(first) position

	First-time PE	First-time PE (no COVID)
Pre-pandemic trend		
April 2008 to February 2020	1.002(1.001-1.002)	1.002(1.001-1.002)
Compared to Baseline period (before Mar 2020)		
March 2020	0.71 (0.67-0.76)	0.70 (0.65-0.74)
April 2020	0.75 (0.59-0.95)	0.69 (0.54-0.88)
May 2020	1.00 (0.90-1.10)	0.97 (0.87-1.07)
June 2020	1.00 (0.90-1.10)	0.98 (0.88-1.09)
July 2020	1.03 (0.93-1.12)	1.02 (0.93-1.12)
August 2020	1.04 (0.99-1.09)	1.03 (0.98-1.09)
September 2020	0.96 (0.89-1.03)	0.94 (0.88-1.02)
October 2020	1.08 (1.00-1.16)	1.02 (0.93-1.12)
November 2020	1.02 (0.94-1.10)	0.92 (0.84-1.01)
December 2020	1.09 (1.06-1.13)	0.95 (0.91-0.99)
January 2021	1.15 (0.99-1.33)	0.96 (0.84-1.11)
February 2021	1.15 (1.05-1.27)	1.06 (0.98-1.15)
March 2021	1.24 (1.17-1.30)	1.21 (1.16-1.26)
April 2021	1.11 (1.07-1.16)	1.10 (1.06-1.15)
May 2021	1.21 (1.14-1.28)	1.20 (1.13-1.28)
June 2021	1.06 (1.01-1.12)	1.06 (1.01-1.11)
July 2021	1.08 (1.01-1.15)	1.05 (1.00-1.11)
August 2021	1.08 (1.02-1.14)	1.05 (1.01-1.09)
September 2021	1.09 (1.02-1.17)	1.05 (0.99-1.11)
October 2021	1.07 (1.02-1.12)	1.03 (0.99-1.08)
November 2021	1.07 (1.01-1.13)	1.03 (0.98-1.08)
December 2021	1.10 (1.05-1.15)	1.04 (1.00-1.08)
January 2022	1.10 (1.02-1.18)	1.01 (0.93-1.09)
February 2022	1.02 (0.98-1.06)	0.96 (0.91-1.01)
March 2022	1.06 (1.02-1.09)	0.96 (0.93-0.99)
April 2022	1.03 (0.99-1.08)	0.93 (0.89-0.97)
May 2022	1.09 (1.05-1.13)	1.04 (1.01-1.08)
June 2022	0.99 (0.94-1.05)	0.92 (0.86-0.99)
July 2022	1.01 (0.98-1.04)	0.91 (0.87-0.94)
August 2022	1.10 (1.07-1.13)	1.04 (1.00-1.08)
September 2022	1.08 (1.01-1.14)	1.01 (0.92-1.10)
October 2022	1.07 (1.04-1.11)	1.01 (0.98-1.04)
November 2022	1.05 (1.00-1.09)	1.00 (0.94-1.05)
December 2022	0.92 (0.89-0.94)	0.85 (0.83-0.88)
January 2023	1.13 (1.09-1.17)	1.07 (1.03-1.11)
February 2023	0.85 (0.82-0.88)	0.80 (0.77-0.83)
March 2023	1.01 (0.97-1.05)	0.94 (0.91-0.97)
April 2023	0.96 (0.93-0.99)	0.90 (0.87-0.94)
May 2023	0.95 (0.91-0.99)	0.91 (0.87-0.96)
June 2023	0.91 (0.86-0.96)	0.89 (0.84-0.95)
July 2023	0.98 (0.95-1.02)	0.96 (0.93-1.00)
August 2023	1.00 (0.97-1.03)	0.97 (0.93-1.00)
September 2023	0.98 (0.93-1.02)	0.94 (0.90-0.98)
October 2023	1.05 (1.02-1.08)	1.01 (0.98-1.04)
November 2023	1.08 (1.03-1.13)	1.04 (1.00-1.09)
December 2023	1.00 (0.95-1.06)	0.96 (0.92-1.00)
January 2024	1.03 (0.99-1.07)	0.99 (0.95-1.04)
February 2024	0.99 (0.95-1.03)	0.97 (0.92-1.01)
March 2024	1.00 (0.96-1.04)	0.98 (0.94-1.03)
April 2024	1.01 (0.97-1.05)	0.99 (0.95-1.03)
May 2024	1.01 (0.95-1.06)	0.98 (0.93-1.03)
June 2024	0.93 (0.90-0.97)	0.90 (0.87-0.94)
July 2024	0.99 (0.95-1.03)	0.94 (0.91-0.98)
August 2024	0.92 (0.89-0.95)	0.89 (0.86-0.93)
September 2024	0.89 (0.85-0.94)	0.87 (0.82-0.92)
October 2024	1.00 (0.95-1.05)	0.96 (0.92-1.01)
November 2024	0.91 (0.87-0.94)	0.89 (0.86-0.93)
December 2024	0.82 (0.78-0.86)	0.81 (0.77-0.85)

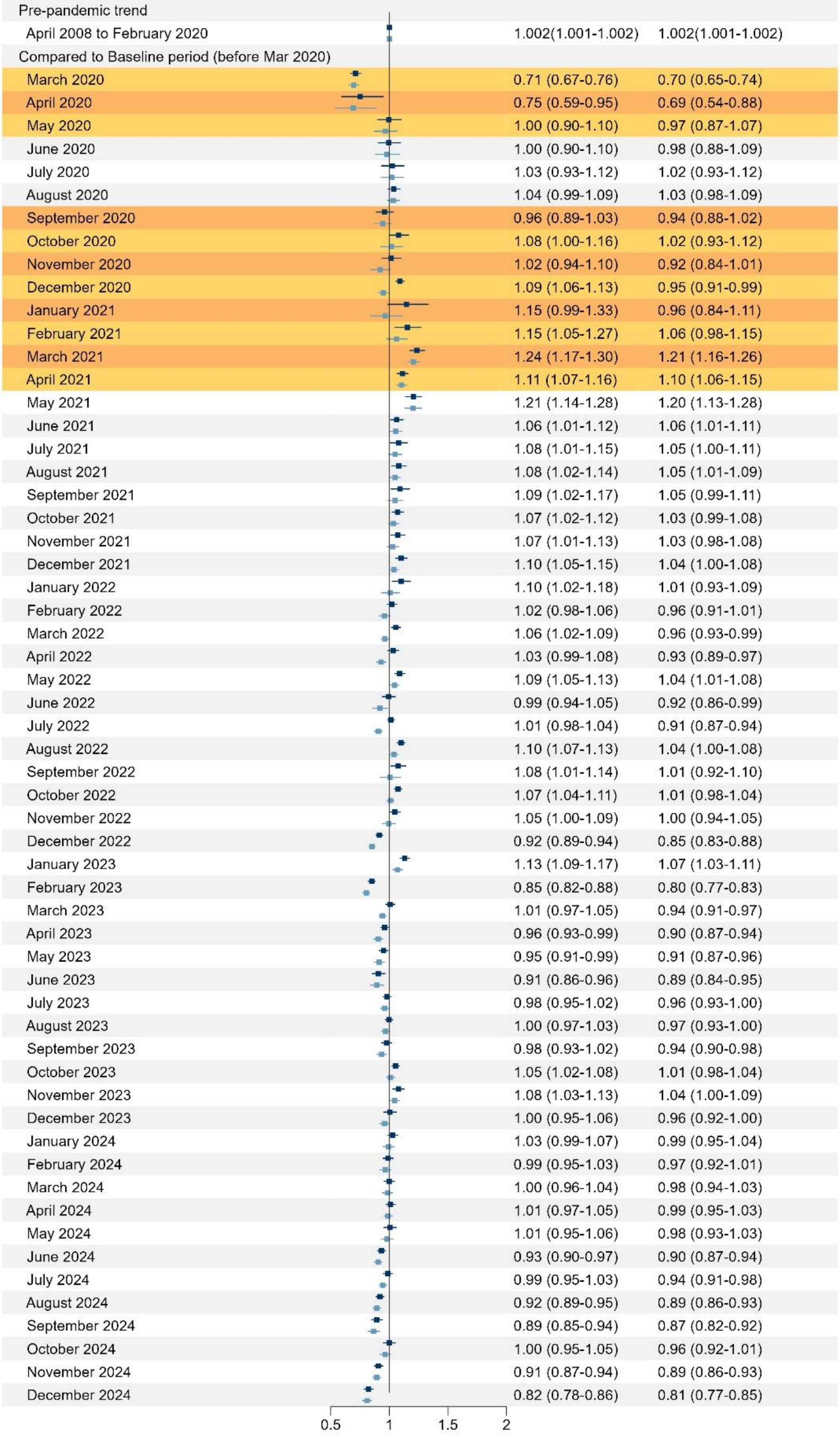

Outcome First-time PE First-time PE (no COVID)

eFigure 13 First-time hospital-recorded PE rates: 42 Integrated Care Boards (ICBs) versus all 44 ICBs, April 2008 – December 2024

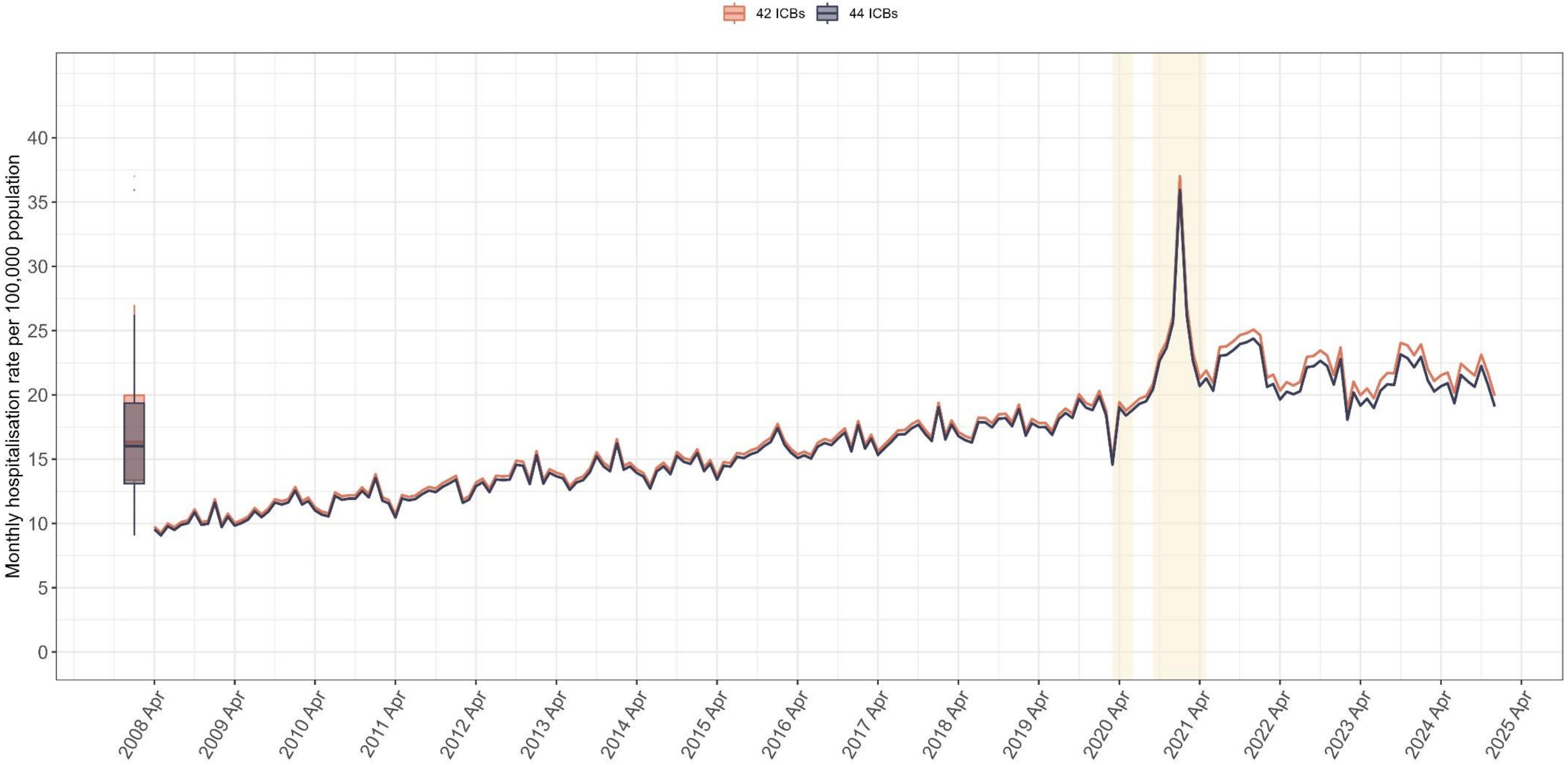

The numerator is the number of first-time admissions to hospital with PE per patient, in any diagnostic position. The denominator is the total baseline population based on mid-year population estimates from the Office for National Statistics. Boxplots show the historical average crude PE rate from April 2008 to December 2024 (median and interquartile range). The shaded area indicates the first and second waves of the coronavirus pandemic in England.

eFigure 14 Effect of the COVID-19 pandemic on first-time PE incidence rates, with and without co-existing COVID-19, comparing post-pandemic months (March 2020 to December 2024) with pre-pandemic trend (April 2008 to February 2020); All 44 ICBs included

	First-time PE	First-time PE (no COVID)
Pre-pandemic trend		
April 2008 to February 2020	1.002(1.002-1.002)	1.002(1.002-1.002)
Compared to Baseline period (before Mar 2020)		
March 2020	0.77 (0.70-0.84)	0.67 (0.63-0.72)
April 2020	1.02 (0.80-1.31)	0.73 (0.59-0.89)
May 2020	1.04 (0.97-1.12)	0.94 (0.87-1.02)
June 2020	1.05 (0.98-1.12)	1.01 (0.94-1.08)
July 2020	1.03 (0.97-1.10)	1.02 (0.96-1.09)
August 2020	1.03 (0.99-1.08)	1.02 (0.97-1.06)
September 2020	1.03 (0.99-1.08)	0.98 (0.93-1.02)
October 2020	1.17 (1.12-1.22)	0.96 (0.91-1.02)
November 2020	1.20 (1.13-1.27)	0.87 (0.82-0.93)
December 2020	1.28 (1.21-1.36)	0.86 (0.81-0.92)
January 2021	1.78 (1.45-2.20)	0.92 (0.82-1.04)
February 2021	1.33 (1.20-1.47)	1.03 (0.98-1.08)
March 2021	1.22 (1.15-1.28)	1.14 (1.11-1.18)
April 2021	1.13 (1.09-1.17)	1.10 (1.07-1.14)
May 2021	1.16 (1.10-1.22)	1.15 (1.08-1.21)
June 2021	1.06 (1.00-1.12)	1.02 (0.98-1.06)
July 2021	1.17 (1.06-1.29)	1.05 (1.01-1.10)
August 2021	1.18 (1.07-1.31)	1.03 (0.99-1.07)
September 2021	1.18 (1.09-1.28)	1.03 (0.98-1.08)
October 2021	1.19 (1.13-1.25)	1.04 (1.00-1.08)
November 2021	1.17 (1.10-1.25)	1.00 (0.97-1.04)
December 2021	1.20 (1.13-1.27)	1.00 (0.96-1.03)
January 2022	1.16 (1.10-1.22)	0.95 (0.91-1.00)
February 2022	1.04 (1.01-1.07)	0.91 (0.88-0.95)
March 2022	1.10 (1.04-1.16)	0.91 (0.88-0.94)
April 2022	1.06 (1.00-1.12)	0.89 (0.86-0.92)
May 2022	1.10 (1.05-1.15)	1.02 (0.98-1.06)
June 2022	1.03 (1.00-1.07)	0.90 (0.86-0.95)
July 2022	1.03 (1.00-1.07)	0.86 (0.84-0.89)
August 2022	1.09 (1.06-1.12)	0.98 (0.95-1.02)
September 2022	1.06 (1.02-1.10)	0.94 (0.88-1.02)
October 2022	1.09 (1.04-1.14)	0.97 (0.93-1.01)
November 2022	1.04 (1.02-1.07)	0.96 (0.92-1.00)
December 2022	0.98 (0.94-1.02)	0.86 (0.84-0.89)
January 2023	1.07 (1.04-1.11)	0.98 (0.94-1.02)
February 2023	0.85 (0.82-0.87)	0.75 (0.72-0.79)
March 2023	0.99 (0.96-1.02)	0.87 (0.83-0.91)
April 2023	0.95 (0.91-0.98)	0.87 (0.83-0.90)
May 2023	0.97 (0.94-1.00)	0.92 (0.88-0.96)
June 2023	0.94 (0.90-0.97)	0.91 (0.88-0.95)
July 2023	0.96 (0.92-1.00)	0.93 (0.88-0.98)
August 2023	0.97 (0.94-1.00)	0.92 (0.89-0.96)
September 2023	0.98 (0.94-1.02)	0.90 (0.86-0.94)
October 2023	1.06 (1.03-1.09)	0.98 (0.95-1.02)
November 2023	1.03 (1.00-1.06)	0.98 (0.95-1.00)
December 2023	0.99 (0.94-1.04)	0.91 (0.88-0.95)
January 2024	1.01 (0.97-1.05)	0.95 (0.90-1.00)
February 2024	0.96 (0.92-1.00)	0.93 (0.89-0.97)
March 2024	0.98 (0.95-1.02)	0.96 (0.92-0.99)
April 2024	0.98 (0.95-1.02)	0.95 (0.91-0.99)
May 2024	1.00 (0.96-1.04)	0.96 (0.92-1.00)
June 2024	0.91 (0.88-0.93)	0.86 (0.83-0.89)
July 2024	0.96 (0.93-1.00)	0.90 (0.87-0.93)
August 2024	0.94 (0.90-0.98)	0.90 (0.86-0.93)
September 2024	0.89 (0.86-0.93)	0.84 (0.81-0.88)
October 2024	0.97 (0.94-1.00)	0.92 (0.89-0.95)
November 2024	0.89 (0.85-0.92)	0.86 (0.83-0.90)
December 2024	0.83 (0.80-0.86)	0.81 (0.78-0.83)

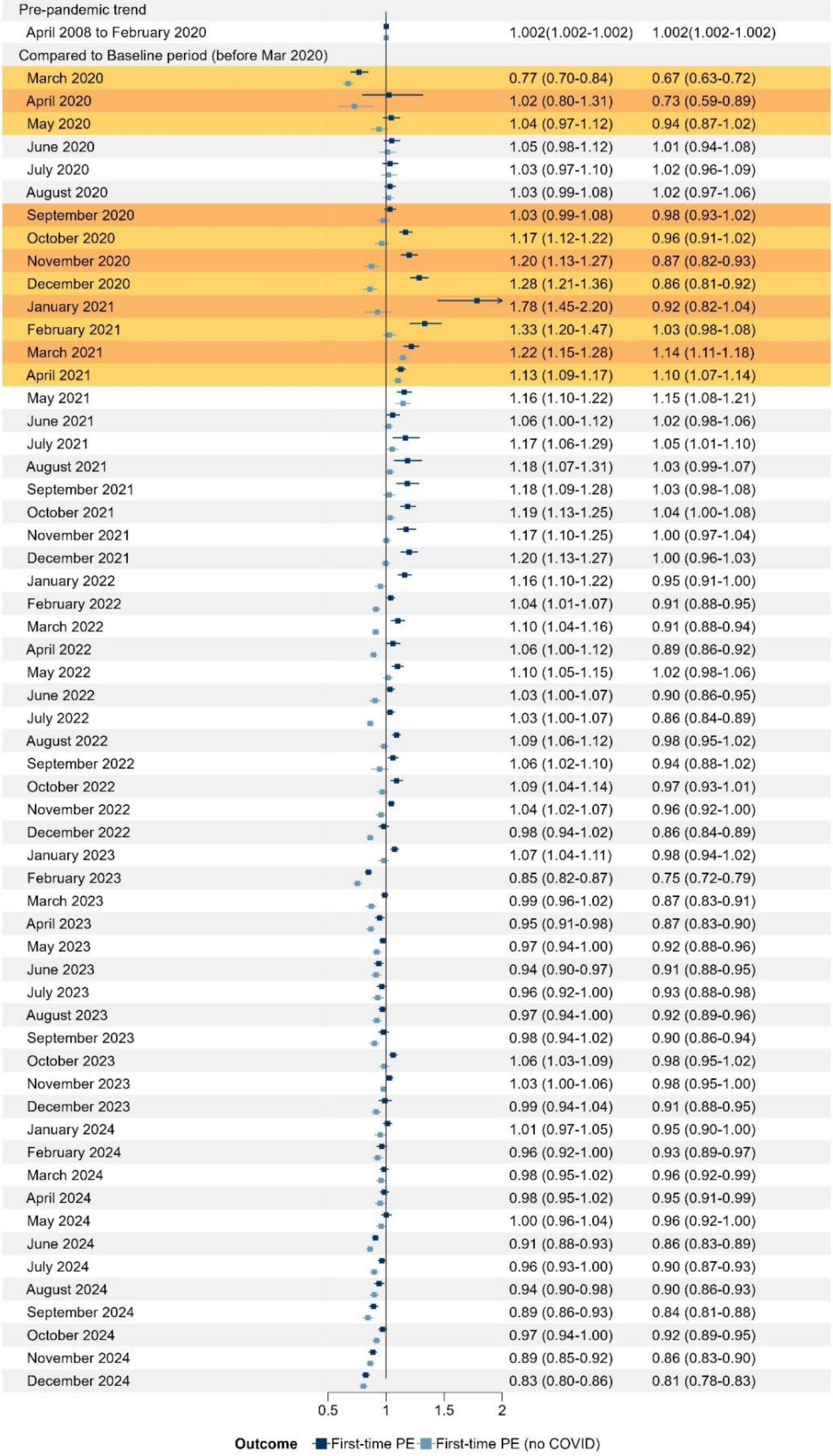

eFigure 15 First-time hospital-recorded PE rates (any position) and first-time hospital-recorded COVID-19 (primary position), April 2008 to December 2024

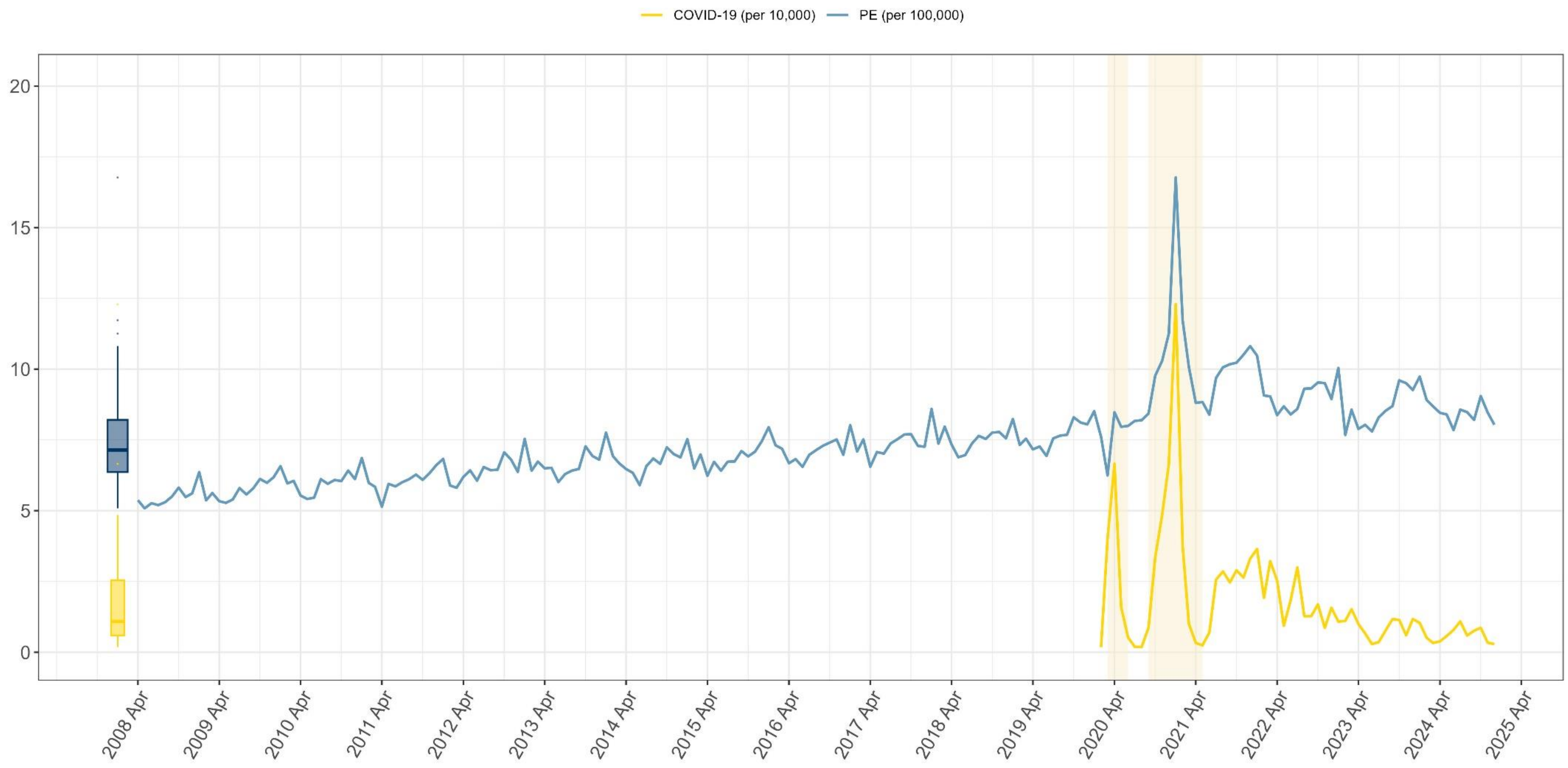

eFigure 16 Joinpoint analysis of first-time hospital-recorded PE: any-position versus primary-diagnosis-only cases/with and without co-existing COVID-19

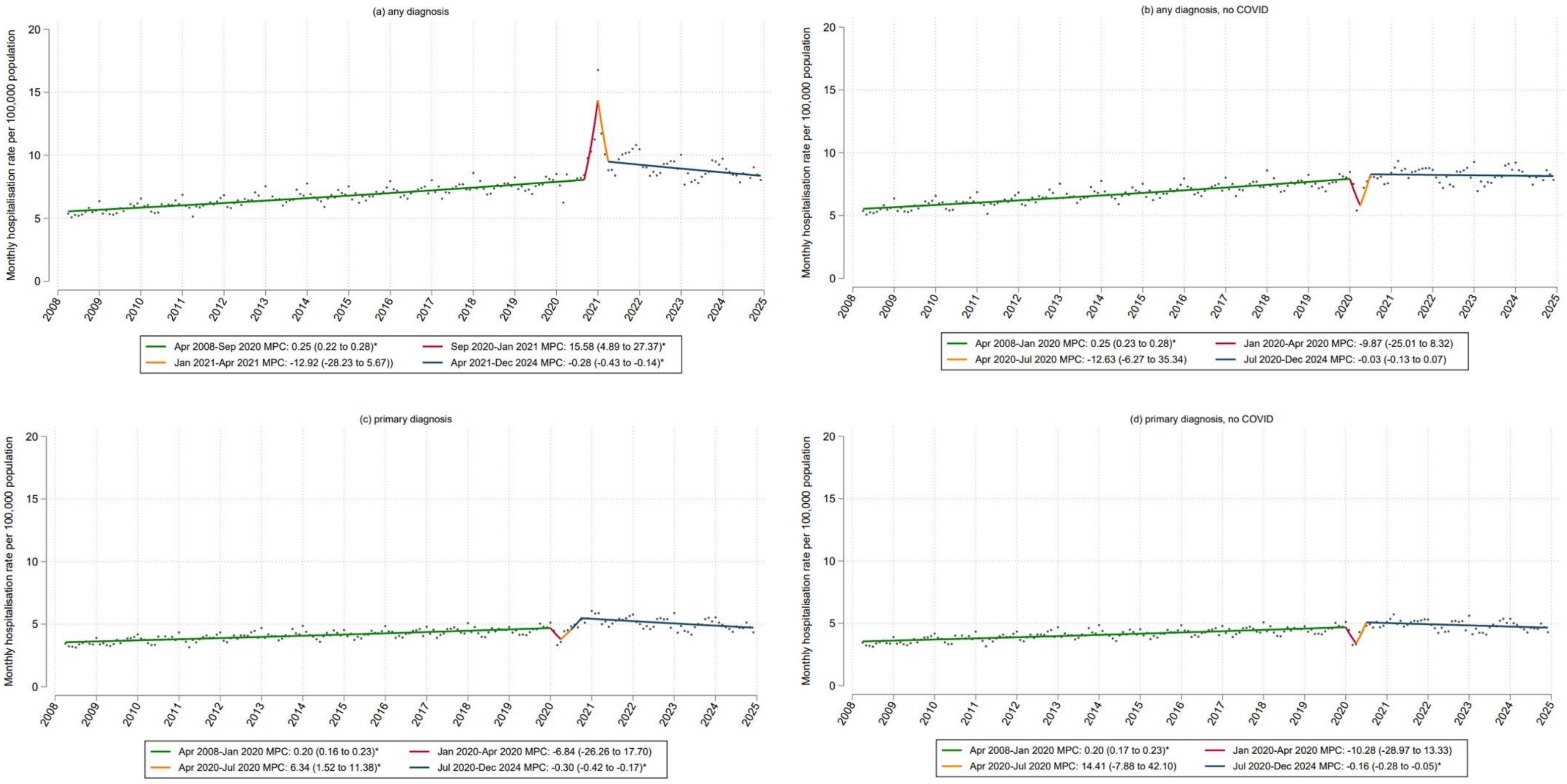

*Indicates that the Monthly Percent Change (MPC) is significantly different from zero at the alpha = 0.05 level. All models were based on the data-driven weighted BIC method. For each analysis, up to five potential joinpoints were allowed; the best-fitting model in each case included three joinpoints.
