# Supplementary material for: Temporal trends in hospital-recorded pulmonary embolism in England before, during and after the COVID-19 pandemic (2008–2024): a population-based observational study
Source: Lancet Reg Health Eur. 2025 Sep 2;58:101433. doi: 10.1016/j.lanepe.2025.101433 (PMC12444491; doi:10.1016/j.lanepe.2025.101433)
Supplement: Supplementary material online tool [file mmc2.zip › lanepe_101433_supplementary material online tool_mmc2.html]

Temporal trends in inpatient-recorded pulmonary embolism (PE) in England

### Time series of counts and age-standardised rates for PE admissions (April 2008 - December 2024)

Demographic:

Metric:

Count
Crude rate
Age-standardised rate

COVID co-diagnosis:

Include
Exclude
