# Supplementary material for: Temporal trends in hospital-recorded pulmonary embolism in England before, during and after the COVID-19 pandemic (2008–2024): a population-based observational study
Source: Lancet Reg Health Eur. 2025 Sep 2;58:101433. doi: 10.1016/j.lanepe.2025.101433 (PMC12444491; doi:10.1016/j.lanepe.2025.101433)
Supplement: Supplementary material STROBE [file mmc3.docx]

STROBE Statement—checklist of items that should be included in reports of observational studies

|  | Item No. | Recommendation | Page  No. | Relevant text from manuscript |
| --- | --- | --- | --- | --- |
| **Title and abstract** | 1 | (*a*) Indicate the study’s design with a commonly used term in the title or the abstract | 1-2 | “Temporal trends … observational study”  “We conducted … early 2023” |
|  |  | (*b*) Provide in the abstract an informative and balanced summary of what was done and what was found | 2 | “We conducted … early 2023” |
| Introduction | | | |  |
| Background/rationale | 2 | Explain the scientific background and rationale for the investigation being reported | 6-7 | "COVID-19 infection increases 2008 to 2024." |
| Objectives | 3 | State specific objectives, including any prespecified hypotheses | 6-7 | “Using national … , and region” |
| Methods | | | |  |
| Study design | 4 | Present key elements of study design early in the paper | 7-10 | “We conducted … in England” |
| Setting | 5 | Describe the setting, locations, and relevant dates, including periods of recruitment, exposure, follow-up, and data collection | 7-10 | “The study period was … per hospital episode” |
| Participants | 6 | (*a*) *Cohort study*—Give the eligibility criteria, and the sources and methods of selection of participants. Describe methods of follow-up  *Case-control study*—Give the eligibility criteria, and the sources and methods of case ascertainment and control selection. Give the rationale for the choice of cases and controls  *Cross-sectional study*—Give the eligibility criteria, and the sources and methods of selection of participants | 7-10 | “Patients were identified as … April 2003” |
|  |  | (*b*) *Cohort study*—For matched studies, give matching criteria and number of exposed and unexposed  *Case-control study*—For matched studies, give matching criteria and the number of controls per case |  | NA |
| Variables | 7 | Clearly define all outcomes, exposures, predictors, potential confounders, and effect modifiers. Give diagnostic criteria, if applicable | 7-10 | “First-time PE-related … in sensitivity analysis).” |
| Data sources/ measurement | 8* | For each variable of interest, give sources of data and details of methods of assessment (measurement). Describe comparability of assessment methods if there is more than one group | 7-10 | *“Data were derived… Population 2013”* |
| Bias | 9 | Describe any efforts to address potential sources of bias | 7-10 | “To minimise bias, only first … missing value” |
| Study size | 10 | Explain how the study size was arrived at | 7-10 | “The study included all .. December 2024 (n = 750,109)” |

Continued on next page

| Quantitative variables | 11 | Explain how quantitative variables were handled in the analyses. If applicable, describe which groupings were chosen and why | 7-10 | “Monthly age-standardised and … European Standard Population 2013.” |
| --- | --- | --- | --- | --- |
| Statistical methods | 12 | (*a*) Describe all statistical methods, including those used to control for confounding | 7-10 | “Interrupted time series analysis… was used for missing data” |
|  |  | (*b*) Describe any methods used to examine subgroups and interactions | 7-10 | “Interrupted time series analysis… was used for missing data” |
|  |  | (*c*) Explain how missing data were addressed | 7-10 | “Interrupted time series analysis… was used for missing data” |
|  |  | (*d*) *Cohort study*—If applicable, explain how loss to follow-up was addressed  *Case-control study*—If applicable, explain how matching of cases and controls was addressed  *Cross-sectional study*—If applicable, describe analytical methods taking account of sampling strategy | 7-10 | “Interrupted time series analysis… was used for missing data” |
|  |  | (*e*) Describe any sensitivity analyses | 7-10 | “To explore … using the joinpoint method” |
| Results | | | | |
| Participants | 13* | (a) Report numbers of individuals at each stage of study—eg numbers potentially eligible, examined for eligibility, confirmed eligible, included in the study, completing follow-up, and analysed | 10-12 | “From April 2008 to December 2024 … described in eFigure 1.” |
|  |  | (b) Give reasons for non-participation at each stage | 10-12 | eFigure1 |
|  |  | (c) Consider use of a flow diagram | 10-12 | eFIgure1 |
| Descriptive data | 14* | (a) Give characteristics of study participants (eg demographic, clinical, social) and information on exposures and potential confounders | 10-12 | Table 1 describes the characteristics of patients with COVID-related PE and non–COVID-related PE |
|  |  | (b) Indicate number of participants with missing data for each variable of interest | 10-12 | The number of participants with missing values for sex, region, and deprivation is reported in Table 1. |
|  |  | (c) *Cohort study*—Summarise follow-up time (eg, average and total amount) |  | NA |
| Outcome data | 15* | *Cohort study*—Report numbers of outcome events or summary measures over time | 10-12 | *Age-standardised temporal trends in first-time PE diagnoses are shown in Figure 1* |
|  |  | *Case-control study—*Report numbers in each exposure category, or summary measures of exposure |  |  |
|  |  | *Cross-sectional study—*Report numbers of outcome events or summary measures |  |  |
| Main results | 16 | (*a*) Give unadjusted estimates and, if applicable, confounder-adjusted estimates and their precision (eg, 95% confidence interval). Make clear which confounders were adjusted for and why they were included | 10-12 | “Comparing observed levels of first-time PE diagnoses in January 2021 with expected levels based on the pre-pandemic trend (i.e., the counterfactual), the IRR was 1.81 [95% CI, 1.46–2.24]. The January 2021 spike was largely accounted for by admissions with co-existing COVID-19 infection. Rates returned to 2019 levels by December 2022.” |
|  |  | (*b*) Report category boundaries when continuous variables were categorized | 10-12 | Age group, sex, region, and deprivation category boundaries are reported in Table 1. |
|  |  | (*c*) If relevant, consider translating estimates of relative risk into absolute risk for a meaningful time period | 10-12 | Table 1 |

Continued on next page

| Other analyses | 17 | Report other analyses done—eg analyses of subgroups and interactions, and sensitivity analyses | 10-12 | Sensitivity analyses are described in the Results and eFigures 8–10, including alternative case definitions and five-year look-back periods. Joinpoint analysis results are reported in eFigure 16. |
| --- | --- | --- | --- | --- |
| Discussion | | | | |
| Key results | 18 | Summarise key results with reference to study objectives | 12-17 | “Following an upward trend from 2008 to 2019 … to pre-pandemic levels by early 2023.” |
| Limitations | 19 | Discuss limitations of the study, taking into account sources of potential bias or imprecision. Discuss both direction and magnitude of any potential bias | 12-17 | We were unable to establish causes of observed trends. PEs treated without admission may not be fully captured. The accuracy of population-based rates is dependent on mid-year ONS estimates. Potential misclassification due to diagnostic coding may remain. |
| Interpretation | 20 | Give a cautious overall interpretation of results considering objectives, limitations, multiplicity of analyses, results from similar studies, and other relevant evidence | 12-17 | Our findings confirm a spike in PE incidence … the need for ongoing surveillance of PE. |
| Generalisability | 21 | Discuss the generalisability (external validity) of the study results | 12-17 | Data from other countries that include the pandemic and post-pandemic periods are needed to clarify the generalisability of our findings |
| Other information | |  | | |
| Funding | 22 | Give the source of funding and the role of the funders for the present study and, if applicable, for the original study on which the present article is based | 3 | NIHR Oxford Biomedical Research Centre. |

*Give information separately for cases and controls in case-control studies and, if applicable, for exposed and unexposed groups in cohort and cross-sectional studies.

**Note:** An Explanation and Elaboration article discusses each checklist item and gives methodological background and published examples of transparent reporting. The STROBE checklist is best used in conjunction with this article (freely available on the Web sites of PLoS Medicine at http://www.plosmedicine.org/, Annals of Internal Medicine at http://www.annals.org/, and Epidemiology at http://www.epidem.com/). Information on the STROBE Initiative is available at www.strobe-statement.org.
